# Leveraging Traditional Ecological Knowledge and Access to Nutrient-Rich Indigenous Foods to Help Achieve SDG 2: An Analysis of the Indigenous Foods of Sauria Paharias, a Vulnerable Tribal Community in Jharkhand, India

**DOI:** 10.3389/fnut.2020.00061

**Published:** 2020-06-02

**Authors:** Suparna Ghosh-Jerath, Ridhima Kapoor, Archna Singh, Shauna Downs, Satabdi Barman, Jessica Fanzo

**Affiliations:** ^1^Indian Institute of Public Health-Delhi, Public Health Foundation of India, Gurgaon, India; ^2^Department of Biochemistry, All India Institute of Medical Sciences, New Delhi, India; ^3^Department of Urban-Global Public Health, Rutgers School of Public Health, Newark, NJ, United States; ^4^Department of International Health, Berman Institute of Bioethics, Johns Hopkins Bloomberg School of Public Health, Johns Hopkins Nitze School of Advanced International Studies, Baltimore, MD, United States

**Keywords:** traditional ecological knowledge (TEK), indigenous foods, indigenous people, micronutrients, undernutrition, tribal

## Abstract

Indigenous food systems of traditional communities are potentially sustainable, have nutrient rich food sources and can enhance dietary diversity. Sauria Paharias, are one of the particularly vulnerable tribal groups of Jharkhand India, who despite residing in rich biodiverse environment and possessing traditional ecological knowledge, lag behind various health, and nutritional indicators. Our study explored their traditional ecological knowledge around indigenous foods (IFs), their routine consumption, access, and nutritive values. A cross-sectional mixed methods study was carried out in 18 villages of Godda district, Jharkhand. Free list of all IFs known to the community was developed using focus group discussions. This was followed by enumerating commonly consumed as well as little or historically used IFs. Following the taxonomic classification of these foods, their nutritive values were searched in literature or food samples were analyzed in accredited laboratories. Reasons for consumption and non-consumption of specific IFs were explored. The community was aware of a large number of IFs (*n* = 193) but only 50% of these were routinely consumed. Rest were either little used or historically consumed. About 47.6% IFs (*n* = 92) were identified using taxonomic classification; of which 87 IFs were classified based on their common names in secondary literature and five food items were collected, herbariums were prepared and identified. Nutritive values were documented for 84 IFs (this included both routinely consumed as well as little used); out of which 55 foods were found to have nutritive values in existing literature and 29 foods were analyzed in laboratory. Many of these IFs were rich in micronutrients like calcium, iron, zinc, folic acid, vitamin A, and vitamin C. Common reasons for preferences or non-consumption of specific IFs included taste, availability, access seasonality, opportunity cost of access and processing time. Promoting adequate intake of commonly accessed nutrient rich IFs and revival of little used IFs while addressing the causes of non-consumption and mainstreaming them into the daily diets could be an effective strategy to increase the intake of micronutrients. Policies focusing on incorporation of nutrient rich IFs into dietary diversification strategies and ongoing supplementary feeding programs can help address malnutrition in the community.

## Introduction

The landforms, vegetation, climatic zones, rivers, and watersheds have worked together for thousands of years to shape and form indigenous land and food systems. These systems comprising of cultivated crops, wild plants, animals, and fungi species, are synergized with the natural environment and adapted to local climatic conditions requiring minimal external inputs (1, 2). Indigenous foods (IFs), an integral part of these systems, can enhance dietary diversity (3–7) and contribute to nutrient intakes. Studies from India and elsewhere report IF intake to be associated with significantly higher intake of protein, fiber, vitamin A, iron, calcium, and riboflavin (8–11). Increased access to IFs collected from forests have resulted in improved household food security (12) and higher diet diversity among children (13). Traditional foods consumed by aboriginal communities of Canada are nutrient rich and contain less fat, sodium, and carbohydrates (especially sucrose) compared to non-indigenous food items consisting chiefly of market foods (14–20).

The indigenous food systems are integrated into the socio-cultural and spiritual activities of the indigenous people who act as vital custodians of this knowledge (1). These people form 5% of the world's population and manage 28% of the world's land surface, that includes some of the most ecologically intact and biodiverse forest areas (1).They have utilized wild plants for nutrition, medicine and natural products while conserving biodiversity (21) and have inherited and interacted with traditional habitats or ancestral territories, retaining their unique cultures and ways of relating to the environment thus acting as vital custodians of the knowledge of these habitats (22).Their rich traditional ecological knowledge (TEK) of food systems could be utilized for promoting sustainable food systems (23) to bolster efforts for amelioration of poverty, hunger, and various forms of malnutrition as well as addressing several of the Sustainable Development Goals (SDGs) (1).Thus, food systems of indigenous peoples could be leveraged to help address SDGs 2 (zero hunger), 10 (reduced inequalities), 12 (sustainable production and consumption), and 13 (climate action), among others.

However, despite this rich knowledge of biodiverse habitats (24), indigenous populations, constituting 15% of the global poor, are disproportionately affected by hunger, food insecurity, and malnutrition. The survival of their indigenous food systems have been threatened by historical phenomena such as colonization, globalization, assimilation, increased urbanization, industrialization of landscapes (25), and other local issues like access to irrigation, land ownership and migration, socio-economic shifts, and local agricultural policies (24, 26, 27). Environmental factors such as overexploitation of major fish stocks, forests and terrestrial wildlife, invasive species usage, pollution and degradation of lands, waterways, and global climate change have also compromised indigenous food systems (25).

The recent renewal of interest toward indigenous communities' food systems, their holistic approach to food generation and resource management can be a tremendous opportunity. The UN Decade of Action on Nutrition (2016–2025) has called for enhancement of sustainable food systems (28). Some of the indigenous systems can be potential models to inform the current debate on creating resilient and sustainable food systems (25).

Jharkhand, a central eastern state of India known for its natural resources and biodiverse agroforestry, is home to several indigenous tribal communities that constitute 26.2% of the state's population. The Sauria Paharias are one of the particularly vulnerable tribal groups (PVTGs) in Jharkhand (29, 30).They are the original inhabitants of the regions of the Rajmahal Hills and the neighboring region of Santhal Paraganas and follow a patrilineal tradition. The hilly ranges and wild dense forests in the region provides a rich biodiverse food environment (31, 32). The community primarily depends on agriculture based livelihoods which they practice on small areas of land, home gardens (*Baris*), and shifting slash and burn hill cultivation (*Kurwa* farming) (33, 34). Simultaneously, forests, rivers, and nearby areas are also sourced for food, either for household consumption or income generation (33). They live in difficult terrains, with low literacy rates, poor access to healthcare facilities, drinking water, and such other basic amenities of life and their income is much below the lowest minimum required for a decent living (35).

Owing to their difficult living conditions and poor access to amenities, the Sauria Paharias are impoverished and lag behind on several health and nutritional indicators despite a rich biodiverse agroforestry environment and TEK (36, 37). The nutritive value of many of their traditional foods is also undocumented. Their current nutritional status could be a result of their impoverished conditions along with new challenges of changing perceptions about traditional diets, resource depletion and decreasing access to natural resources resulting in a narrowing food basket (38, 39). Their biodiverse environment could be utilized in synchrony with TEK to foster propagation of IFs by reinforcing historical knowledge with scientific inputs for the nutritional well-being of the community (23). This could offer manifold benefit by addressing both malnutrition and ultimately, helping to strengthen the resilience of the socio-cultural and environmental ecosystem (40).

The present study was undertaken to identify the indigenous foods of Sauria Paharia tribal community of Jharkhand, assess the dietary preference of the community toward specific IFs, conduct the taxonomic classification of IFs and estimate their nutritive values. The study further explored the status of access to different IFs to identify gaps in knowledge and actual access and the reasons thereof. The findings will help us gauge the potential of IFs to address nutritional deficiencies and inform us on ways of improving their access with the view to address sustainable development.

## Materials and Methods

### Study Design

This was a cross-sectional mixed-methods study conducted to explore the traditional ecological knowledge about IFs of the Sauria Paharia community and estimate the nutritive values of these foods with a longitudinal component of seasonality to assess access to these foods in two different seasons.

**Figure 1:**
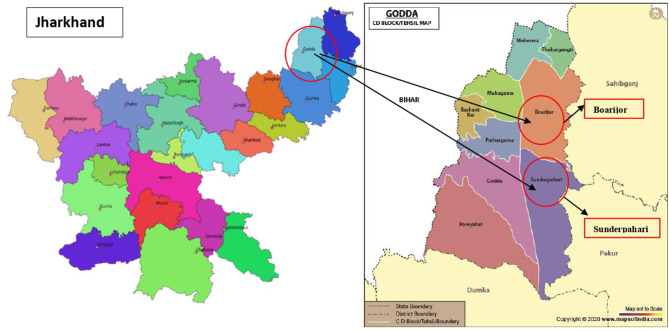
Selection of Sunderpahari and Boarijor blocks in Godda district, Jharkhand.

The study was carried out in 18 selected villages of Sunderpahari and Boarijor blocks (a block is defined as an administrative sub division of a district) in Godda district of Jharkhand, India (Figure 1).These villages (9 villages per block) were identified using probability proportional to size sampling, details of which are reported elsewhere (41). This work is part of a larger study documenting the role of IFs consumed by tribal groups of Jharkhand in addressing food security and dietary diversity among women and children. The data was collected between March 2018 and January 2019; multiple visits were made to capture the diversity of foods consumed during each season. The core research team included nutritionists, anthropologist and a biochemist. In addition to the core research team, the study team also included trained local field workers fluent in the native Paharia dialect for facilitating the discussions. Out of the 18 villages, qualitative data was collected from 11 villages till point of saturation and the quantitative data on access to food was collected from all 18 villages inhabited by Sauria Paharia community (Table 1).

**Table 1:** List of villages in Sunderpahari and Boarijor blocks of Godda district, Jharkhand.

**Block 1. Sunderpahari**	**Block 2: Boarijor**
Village 1 Tasaria^*^	Village 1 Teletok
Village 2 Kusumghati^*^	Village 2 Kusumghati^*^
Village 3 Paharpur^*^	Village 3 Lutibahiar^*^
Village 4 Chewo^*^	Village 4 Bara-amra^*^
Village 5 Longodih^*^	Village 5 Kortica^*^
Village 6 Nadgoda^*^	Village 6 Adro
Village 7 Kheribari	Village 7 Lohatamba
Village 8 Dahubera	Village 8 Chota Dumarhir
Village 9 Kusumaha	Village 9 Rajapokhar^*^

* *Both qualitative and quantitative data collected*.

### Study Procedures

#### Qualitative Methods

Qualitative enquiries were conducted using focus group discussions (42) supported by a village transect walk (43).

#### Transect Walk

As part of the qualitative enquiries and till the point of data saturation, transect walks were performed in 11 out of 18 villages to observe their locations, the landscape, main land uses and distribution of resources in the context of food access including market access. This was conducted by the core team assisted by a village elder or the community health and nutrition worker of the village.

#### Focus Group Discussions: Free Listing and Preferences for Specific Indigenous Foods

Focus group discussions (FGDs) were used to elicit a detailed description of IF availability, access, and utilization in the community. The community health worker and the village head were informed in advance about the FGD. They were asked to inform the community members about the scheduled FGD. In order to ensure adequate representation from different age groups and stakeholders in the community, adult women, adult men, the elderly (men and women), community health, and nutrition workers and community leaders were invited to participate. Mothers were especially invited by the core team and the community health workers to attend because they were mainly responsible for food preparation and feeding their families. The FGDs were held in accessible areas such as *anganwadi* centers (community based centers for women and children) or in open areas in the villages. The FGDs that used pre-tested FGD guides facilitated a free listing exercise to identify the range of foods including IFs consumed by the community. Information on the local names of plant or animal foods and their characteristics such as availability, seasonality, and habitats were also documented. The various foods identified were then categorized under different food groups based on their edible parts/parts consumed (leaves, roots and tubers, vegetables, seeds, fruits, etc.). Participants were also asked to list IFs that are popular, preferred, little used, and historically consumed. The perceptions on the reason why specific IFs were preferred or little used (e.g., availability, accessing/production, and/or taste) were also assessed using the FGD guide.

#### Pairwise Ranking

Pairwise ranking method (44) was used to assess preferences for specific IFs over others under a specific food group. For this, after the free listing exercise, the participants were asked to identify 4–5 preferred IFs as well as non-IFs, within each food group and in a particular season; for example, green leafy vegetables (GLVs), cereals, vegetables, etc. These preferences were based on criteria of taste and availability of particular food items. These preferred foods items were then tabulated as a matrix on a flip chart. An example of pairwise ranking of indigenous vegetables is provided in Table 2.

**Table 2:** Example of Pairwise ranking of indigenous varieties of vegetables.

	***Simbi***	***Kundri***	***Jhingli***	***Ziruli***	***Kachnar Flower***	***Sonpu Flower***
Simbi	X	*Simbi*	*Simbi*	*Simbi*	*Simbi*	*Simbi*
Kundri		*X*	*Kundri*	*Kundri*	*Kundri*	*Kundri*
Jhingli			X	*Ziruli*	*Kachnar Flower*	*Sonpu Flower*
Ziruli				X	*Ziruli*	*Ziruli*
Kachnar Flower					X	*Kachnar Flower*
Sonpu Flower						X

*Simbi-5; Kundri-4; Ziruli-3; Kachnar flower-2; Sonpu flower-1; Jhingli-0*.

#### Quantitative Methods

Different quantitative methods used for assessing access to different IFs, their identification and nutrient analysis are as follows:

**Food Access:** Pre-tested tools were used to elicit information on different foods accessed by the community in two crop cycles i.e., (i) summer and (ii) monsoon and/or winters. These included an agriculture diversity tool and a market survey tool. A detailed agriculture diversity (41, 45) tool (was administered on a sample of 60 households with 3–4 households each conveniently selected from all 18 villages. The tool captured information on variety of foods grown, gathered and accessed as well as animals raised/hunted for food. Information was elicited on type of food variety (indigenous^1^/non-indigenous^2^ or hybrid^3^) accessed as well as their sources such as agricultural fields, burnt patches of forest land (*kurwa*), kitchen gardens (baari), nearby forests, and water resources such as man-made ponds, creeks or dams. Market surveys (48) were also carried out to collect information on different foods available as well as their prices in the local weekly market (*haat*) with special emphasis on agrarian foods and their varieties. Surveys were conducted by the local field team in 5 local markets that were routinely visited by the study villages as reported during the village transect.

#### Identification of Food Samples

Based on the free listing activity done through FGDs, a list of commonly consumed IF items were compiled (including cereals, legumes/pulses/seeds, roots and tubers, vegetables, GLVs, fruits, mushrooms, and animal foods). A literature search based on the common names of the IFs and their photographs (in some cases) was done for taxonomic classification with key inputs from Plant Taxonomists and Botanists. Some food samples whose taxonomic classifications were not available, were collected based on seasonal availability and herbariums were prepared. Standard protocols were followed for herbarium preparation (49) and these herbariums were sent to an expert team at Botanical Survey of India (BSI), Kolkata for taxonomic classification. Details of the sample collection methodology for herbarium preparation is provided elsewhere (41).

#### Nutrient Analysis

After the taxonomic classification of IFs, existing food composition tables including the Indian Food composition tables (50) and other literature sources (9, 51, 52) were searched to identify pre-existing information on their nutritive values. Food samples with missing or no available nutritive values in the secondary literature were collected by the field team subject to their seasonal availability and sent to a NABL (National Accreditation Board for Testing and Calibration Laboratories) accredited laboratory for nutrient analysis. The sample collection was carried out following the standard protocol developed as part of the larger study (41) and nutrient analysis was done according to standard reference protocols developed as part of the study in consultation with the laboratory. The parameters analyzed included energy, protein, carbohydrate, fat, dietary fiber, vitamin A (as beta-carotene), vitamin C, vitamin B1, B2, iron, calcium, zinc, folic acid, and phosphorous. Energy, fat, and dietary fiber were analyzed using gravimetric method, protein via titrimetric method while carbohydrate was measured by difference. The vitamins were assessed using High-Performance Liquid Chromatography (HPLC) and the minerals by Inductively Coupled Plasma Mass Spectrometry (ICP-MS). The analyte values were reported per 100 g of edible weight. The details of the methods used for specific nutrients and the limit of quantification are provided in Supplementary Table 1. Around 10% of the samples were sent simultaneously to another laboratory for analysis as a quality check measure to confirm the accuracy and fidelity of the values obtained at the primary analytical laboratory.

### Data Analysis

All the FGDs were recorded and transcribed from *Paharia* to Hindi by the local field team. The Hindi transcripts were then translated to English. The text of the FGDs was analyzed using thematic analysis. Atlas.ti version 8 was used for coding and analyzing the content of the transcripts. Codes were created to help identify similar sub-themes under broad themes. The data were used to generate a compiled free list of all the IFs currently, as well as historically, consumed by the community along with their habitat and seasonality. Other themes related to their popularity and frequency of use were also examined. The taxonomic classification of IFs that were available either from secondary sources or done as part of the study was added to this list^4^. From the free list, a short list of commonly consumed IFs was made. These were the ones currently consumed by the community and were available for a significant duration round the year. The information from pairwise ranking was used to create comparisons and scoring of commonly consumed foods under each food group and preferred over the others. List of little consumed foods was compiled that included rarely consumed foods as well as foods that were historically consumed by previous generations. The agricultural diversity and market survey data were used to develop a comprehensive list of foods (indigenous, non-indigenous, and hybrid varieties) accessed within the community during two seasons in the past 1 year. The foods (including indigenous indigenous/non-indigenous/hybrid) accessed, were further categorized according to their place of procurement and seasonality. The nutritive value of IFs were utilized to prepare a list of micronutrient rich IFs under commonly consumed and little used ones.

### Ethics Approval

The study was conducted according to guidelines laid down in Declaration of Helsinki (53), and all procedures involving humans in this study were approved by the Institutional Ethics Committee at Indian Institute of Public Health-Delhi, Public Health Foundation of India and All India Institute of Medical Sciences, New Delhi. Administrative approvals from authorities at district level were also taken. Cluster level consents from the village leaders were obtained before conducting the data collection. Written informed consent was obtained from all participants who were literate. Third-party witnessed verbal consents were obtained from illiterate participants. All participants were informed that the FGDs were going to be recorded and that no personal information would be used in any of the study reports. Permission was taken for pictures to be taken during the FGDs.

## Results

Among the 11 Sauria Paharia villages where village transects were conducted, almost all villages were nestled amidst the hilly terrains of the selected blocks. The villages typically consisted of rows of houses. Food was accessed from different sources like the small kitchen garden (*ghar-bari*) adjoining the houses; comparatively larger plots of land used for growing crops and vegetables at the same elevation as house (*bari*); and from farm land which were at a lower elevation. Most but not all villages accessed forests for gathering foods as well as doing slash and burn farming (*kurwa*) and consuming the produce. Some villages accessed fish and water animals from the river flowing within 1 km of the village or mountain spring within 2 km. There were small shops within the villages that sold basic food stuffs like salt, oil, some grains, and packaged snacks and vendors also frequented the villages selling fruits, and packaged and ready to eat snacks. All villages had access to Anganwadi centers that provided supplementary foods to children and pregnant and lactating women and schools that distributed mid-day meals. The public distribution system shops distributing subsidized cereals and sugar were within 5 km of each village. The villagers also accessed weekly markets which were located within 5 km.

**Figure 2:**
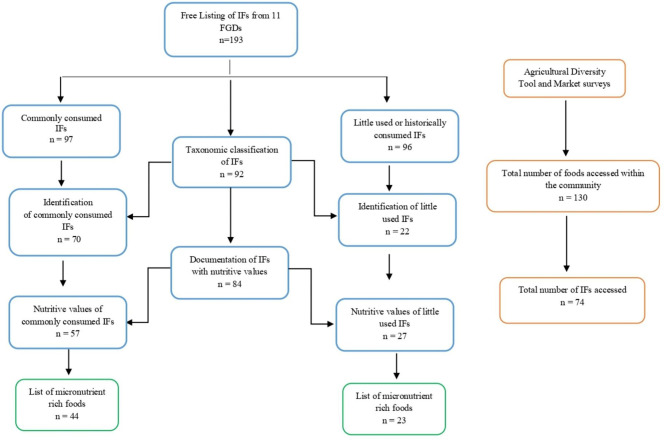
Systematic data collection with number of IFs studied at each stage of the study.

Figure 2 summarizes the systematic approach adopted and total number of IFs assessed at each stage of data collection using qualitative enquires and quantities surveys.

### Traditional Diets of Sauria Paharia Tribes

#### General Eating Pattern

Based on the FGDs conducted, the community reported consuming a rice based diet with the majority of the households consuming two main meals a day. Meals consisted of rice along with sautéed or curried GLVs or roots and tubers or sometimes pulses and flesh foods (meat, poultry, egg, or fish). The respondents reported consuming seasonal fruits especially wild fruits, mushrooms during rainy season, and hunted flesh foods (very occasionally) like wild pigs, birds and rodents, and collected fish and water animals like snails and crabs. A large variety of GLVs, other vegetables and roots and tubers were reported in all the discussions. Some of these were grown in kitchen gardens, some were collected from cultivated lands (where they grew as weeds), forests, and open access areas, and some were procured from the local markets. Some of the food items such as GLVs, fruits, and mushrooms were sun-dried for storage and preservation. Milk and milk products were reported to be rarely consumed by the community.

Table 3 provides a list of all the IFs mentioned by the study participants with details of the parts consumed (in case of plants), their primary sources from where they were harvested, gathered, or collected and the season in which they are available. More than one part of some of the listed IFs were reportedly consumed. The edible part of Colocasia (***Makedi****; Colocasia antiquorum)* for example, included both its leaves and root. A total of 10 such foods were found with more than one edible part. Based on this, the free listing activity provided us with a total of 193 indigenous food items. This included 16 cereals (8.3%), 6 pulses (3.1%), 36 GLVs (18.7%), 14 other vegetables (7.3%), 23 roots and tubers (11.9%), 19 fruits (9.8%), 33 mushrooms (17.1%), 43 flesh foods (22.3%) and 3 miscellaneous items (1.5%). Apart from the IFs, the non-indigenous or hybrid varieties of several foods were also mentioned by the community which are not documented in the table.

**Table 3:** IFs with taxonomic classification, part(s) consumed, place of procurement and seasonality.

**S no**.	**Local (Paharia) name**	**Common name**	**Scientific classification**	**Scientific name**	**Part consumed**	**Accessed/grown**	**Seasons (summer/monsoon/winter/all seasons)**
1–11.	Swarna^#^, Bhadai^*$*^, Sarda^#^, Arwa^*$*^, Sarap^*$*^, Jesori, Bahyar^*$*^, Bangla Bhat^*$*^, Hariya^*^, Banagal^*^, Lal Dhan^*^	Varieties of Rice	✓	*Oryza sativa* L.	Grain	Field	Different varieties grew in different seasons. Superscript provides details on seasonality
12.	*Makai/Gangi (Desi)*	Maize	✓	*Zea mays* L.	Cob	Field, Baari	All season
13.	*Bajra/Shishua (Desi)*	Pearl millet	✓	*Pennisetum typhoideum* Rich.	Kernel	Field	All season
14.	*Kodo/Mandua (Desi)*	Finger millet	✓	*Eleusine coracana* (L.) Gaertn.	Grain	Field	All season
15.	*Jowar (Desi)*	Sorghum	✓	*Sorghum vulgare* Pers.	Grain	Field	All season
16.	Gondli^*****^	-	×	*-*	Grain	Field	-
17.	*Jatta usro/Ghangra*	Cowpea, brown	✓	*Vigna catjang* (L.) Walp.	Seed and vegetable	Field	All season, Winter (vegetable)
18.	*Eso Usro*	Cowpea, white	✓	*Dolichos catjang* Burm.f	Seed	Field	All season
19.	*Kakro/Suthro/Suthri*	Rice bean	✓	*Phaseolus calcaratus* Roxb.	Seed	Field	Winter
20.	*Kurthi*	Horse Gram, whole	✓	*Dolichos biflorus* L.	Seed	Field	Monsoon and/or Winter
21.	*Khesari (Desi)*	Khesari	✓	*Lathyrus sativus* L.	Seed and leaves	Field, Market	Monsoon and/or Winter
22.	*Kusa*	-	✓	*Mucuna pruriens* (L.) DC.	Seed	Field	-
23.	*Makedi*	Colocasia leaves	✓	*Colocasia antiquorum* Schott	Leaves and root	Forest	Monsoon
24.	*Komo* **(IIPHD-006)**	Koinaar leaves	✓	*Bauhinia purpurea* L.	Leaves	Forest, Open space	Summer
25.	*Daav Ghasi*	Kantha leaves	✓	*Euphorbia granulate* Forssk.	Leaves	Field (wild)	Monsoon
26.	*Lol Ghasi*	Bottle gourd leaves	✓	*Lagenaria vulgaris* Ser.	Leaves	Baari, Open space (wild)	Summer
27.	*Tisso Ghasi*	Mata leaves	✓	*Antidesma diandrum* (Roxb.)	Leaves and fruit	Forest	All season
28.	*Chilo Ghasi/Kodgo*	Sinduar leaves	✓	*Celosia argentia* L.	Leaves	Wild	Summer
29.	*Aloo Ghasi*	Potato greens	✓	*Solanum tuberosum* L.	Leaves	Market	Winter
30.	*Sanjhori*	Drumstick	✓	*Moringa oleifera* Lam.	Leaves and flower	Baari	All season
31.	*Adro Ghasi/Margi adro*	Amaranth leaves	✓	*Amaranthus spinosus* L.	Leaves	Baari(wild), Open space	Summer
32.	*Gochi Ghasi*	Ponnaganni	✓	*Alternanthera sessilis* (L.) R.Br. ex DC.	Leaves	Baari (wild), Open space	Summer
33.	*Berbayo Ghasi*	Kena leaves	✓	*Commelina benghalensis* L.	Leaves	Baari(wild)	Rainy
34.	*Kondi Ghasi* **(IIPHD-009)**	Dhurup leaves	✓	*Leucas lavandulifolia* Sm.	Leaves	Open space (wild)	Autumn
35.	*Boot Ghasi*	Bengal gram leaves	✓	*Cicer arietinum* L.	Leaves	Baari	Winter
36.	*Naolo Ghasi* **(IIPHD-010)**	-	✓	*Trianthema portulacastrum* L.	Leaves	Baari (wild)	Autumn
37.	*Pakkedi*	Banyan leaves	✓	*Ficus benghalensis* L.	Leaves and fruit	Forest/Open space	Summer
38.	*Junjuni/Amadro*	Sunsuni leaves	✓	*Marsilea minuta* L.	Leaves	Weed, field	All season
39.	*Nasni Ghasi*	Garlic leaves	✓	*Allium sativum* L.	Leaves	Baari, Market	-
40.	*Pondka Saag*	Malabar spinach	✓	*Basella rubra* L.	Leaves	Field	Autumn
41.	*Zaraael*	Ziruli leaves	✓	*Indigofera cassioides* DC.	Leaves and flower	Wild	Monsoon and/or Winter
42.	*Sonpu*	-	✓	*Crotolaria juncea* L.	Leaves and flower	Baari	Winter
43.	*Chiniya Saag*	-	✓	*Brassica campestris* L.	Leaves	Forest	Monsoon and/or Winter
44–57.	Gobero Adro, Pusre Adro, Gutni, Chiroti Saag, Acchadro, Aradiyo Ghasi, Ursudi Ghasi, Jonya Ghasi, Kannasedi saag, Bodo Ghasi, Dababotri Ghasi, Kotua Saag, Madhari Saag, Manaadro Saag^*****^	Unidentified folk species of GLVs			Leaves	Forest, BaariOpen space (wild)	Monsoon-Winter
58.	*Bir Karela*	Bitter gourd	✓	*Momordica charantia* L.	Vegetable	Baari	All season
59.	*Simbi*	Field beans, tender	✓	*Dolichos lablab* L.	Vegetable	Baari and Market	Winter
60.	*Jhingli (Desi)*	Ridge gourd	✓	*Luffa acutangula* (L.) Roxb.	Vegetable	Baari	Monsoon and/or Winter
61.	*Maas ardo /Karu/Baans*	Bamboo tender	✓	*Bambusa vulgaris* Schrad.	Vegetable	Forest/Open space	Monsoon
62.	*Kokri*	Spine gourd	✓	*Momordica dioica* Roxb.	Vegetable	Baari	Monsoon and/or Winter
63.	*Kundri*	Kovai	✓	*Coccinia cordifolia* (L.) Cogn.	Vegetable	Baari, forest	All season
64.	*Kachna Phool* **(IIPHD-004)**	Kachnar flower	✓	*Bauhinia variegata* L.	Flower	Forest	Winter
65.	*Beralli*	-	✓	*Dioscorea* spp.	Root and vegetable	Open space	Summer
66.	*Pinra/Pindra*	-	✓	*Flacourita indica* (Burm f.) Merr.	Vegetable and root	Forest	Summer
67.	*Zarkunda*	Ashgourd	✓	*Benincasa hispida* (Thunb.) Cogn.	Vegetable	Baari	Winter
68.	*Misrikand*	Yam beans	✓	*Pachyrhizus erosus* (L.) Urb.	Root	Field, Market	Summer
69.	*Lal Aloo*	Red Potato	✓	*Solanum tuberosum* L.	Root	Field, Market	Summer
70.	*Nappe/Nappa*	-	✓	*Dioscorea pentaphylla* L.	Root	Forest	Monsoon and/or Winter
71.	*Singla*	Ole	✓	*Amorphophallus paeoniifolius* (Dennst.) Nicolson	Root	Forest	Monsoon and/or Winter
72–87.	Jattali, Porniali, Churka/Churke, Taalko, Chalangan/Chalango, Pandgo/Pangdro, Chambiyali, Gumalli, Alli, Isaha Alli, Aeli, Gomo, Chalko, Igzol, Panne, Keso ^*****^	Unidentified folk species of roots and tubers			Root	Forest	Autumn-Summer
88.	*Ambad Pupu*	Ambada	✓	*Spondias mangifera* Wild.	Fruit	Forest	Summer
89.	*Madgi*	Mahua, ripe	✓	*Madhuca indica* J.F.Gmel.	Fruit	Open space	Summer
90.	*Kero/Keero Toso*	Marking nut (kernel)	✓	*Semecarpus anacardium* L.f.	Fruit	Forest	Winter
91.	*Ilkarpu/Ber*	Zizyphus	✓	*Zizyphus jujube* Mill.	Fruit	Forest	Winter
92.	*Telo/Kenda/Kaanda/Kendu*	Tumki	✓	*Diospyros melanoxylon* Roxb.	Fruit	Forest	Summer
93.	*Talmi*	Palmyra fruit, ripe	✓	*Borassus flabellifer* L.	Fruit	Open space	Summer
94.	*Pusra*	Kusum	✓	*Schleichera* oleosa (Lour.) Merr.	Fruit	Forest	Summer
95.	*Dahu/Tisgo Chagzo*	–	✓	*Artocarpus lakoocha* Roxb.	Fruit	Forest	Summer
96.	*Piyaara*	–	✓	*Buchanania lanazan* Spr.	Fruit	Forest	All season
97.	*Haani* **(IIPHD-003)**	–	✓	*Ficus exasperata* Vahl.	Fruit	Forest	Autumn
98.	*Bel/Otte*	Wood apple	✓	*Aegle marmelos* (L.) Correa	Fruit	Open space	Summer
99.	*Dumari/Dumaari/Dungri*	–	✓	*Ficus glomerata* Roxb.	Fruit	Forest	Monsoon
100–104.	Zara Aeli, Anni, Kaisge, Kaita, Dhela^*****^	Unidentified folk species of fruits			Fruit	Forest, Open space	Monsoon
105.	*Singhi Machhli*	Singhi	✓	*Saccobranchus fossilis*	Meat	Water bodies	All season
106.	*Potha/Pothi Machhli*	Puti	✓	*Burbus* spp.	Meat	Water bodies	All season
107.	*Ghongri*	Snail	✓	*Pila globoasa*	Meat	Water bodies	Summer and Monsoon
108.	*Maako/Jhinuk*	Mussels	✓	*Margaritifera margaritifera*	Meat	Water bodies	Summer and Monsoon
109.	*Magur/Mangri Machhli*	Walking catfish	✓	*Clarias batrachus*	Meat	Water bodies	All season
110.	*Boari Machhli*	Wallago	✓	*Wallago attu*	Meat	Water bodies	All season
111.	*Gacchi Machhli*	Freshwater eel	✓	*Anguilla Anguilla*	Meat	Water bodies	All season
112.	*Tengra/Tonger Machhli*	Catfish	✓	*Mystus vittatus*	Meat	Water bodies	All season
113.	*Silong Machhli*	Silhan	✓	*Silonia silondia*	Meat	Water bodies	All season
114.	*Gadai/Gowari Machhli*	Snake head fish	✓	*Channa punctate*	Meat	Water bodies	All season
115.	*Chala/Chalgo Machhli*	Silver razor belly minnow	✓	*Salmostoma acinaces*	Meat	Water bodies	All season
116.	*Baale Machhli*	Bele fish	✓	*Glossogobius giuris*	Meat	Water bodies	All season
117–129.	Zimali, Eherchali, Chachara, Chuchi, Mustura, Gotti, Doke, Mitra, Erke, Banjakudi, Chete, Chatarkati, Jambuchett Machhli^*****^	Unidentified folk species of fishes			Meat	Water bodies	Monsoon
130.	*Chetado ka anda*	Eggs of red ants	✓	*Aceophylla smaragdina*	Meat	Forest	All season
131.	*Gilhari ka maas*	Squirrel meat	✓	*Sciuridae*	Meat	Forest	All season
132.	*Moosa*	Field's rat	✓	*Rattus argentiventer*	Meat	Forest	Monsoon and/or Winter
133.	*Chuwa*	Peacock	✓	*Pavo cristatus*	Meat	Forest	Monsoon and/or Winter
134.	*Mahalo/Mahala*	Wild cat	✓	*Felis catus*	Meat	Forest	All season
135.	*Jangli suar/Kissu*	Pig	✓	*Sus scrofa*	Meat	Forest	All season
136.	*Kissa/Chitru*	Porcupine	✓	*Erethizon dorsatum*	Meat	Forest	All season
137.	*Jangli murgi*	Fowl	✓	*Galloanserae*	Meat	Forest	All season
138.	*Teetar*	Partridge	✓	*Gray francolin*	Meat	Forest	All season
139.	*Pervan*	Pigeon	✓	*Columba livia domestica*	Meat	Forest	All season
140.	*Bater*	Quail	✓	*Coturnix coturnix*	Meat	Forest	All season
141.	*Edru/tota*	Parrot	✓	*Psittacine*	Meat	Forest	All season
142–147.	Tirikado (Chidiya), Pura (Chidiya), Tenga, Kafo, Tura, Oda ^*****^	Unidentified folk species of birds			Meat	Forest	All season
148–150.	Haubudu, Teni, Isge^*****^	Unidentified folk species of insects			Honey	Forest	All season
151–183.	Makko/Maango, Kero/Aero/Khero, Parango, Baado, Endro/Adro/Edro, Naango, Baansosu, Tele-kuto/Telo-kuti, Kaijo, Takna, Tero, Baalco, Ado, Korho/Orho, Kerusudo, Patanglo/Pattangulo/Putka, Pattodi/Pittodi, Chaandi, Jambuaajo, Chaariyoni, Gobroosu, Baanipoto, Kuttapuda, Bandho aero, Bagdoto, Jhinganu/Jhingan, Nalo Osu, Baloosu, Mokro/Mokeroosu, Patla aero, Isuno, Jinpro aero, Ganda budi^*****^	Unidentified folk species of mushrooms			Upper part of mushroom (cap) and stem	Forest	Monsoon

*Text in Italics represents Paharia names*.

*Text in bold represents Voucher/Specimen number of the herbariums identified by Botanical Survey of India, Central National Herbarium, Howrah-711103*.

***** *Taxonomic classification not available*.

Seasonality:

# All seasons;

$ winter season;

* *Summer season*.

### Identification and Taxonomic Classification of Indigenous Varieties of Foods

**Figure 3:**
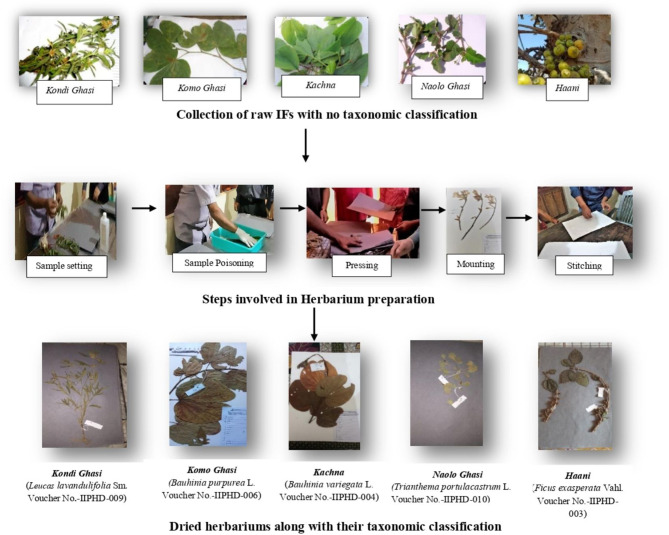
IFs identified from herbarium. *Text in italics and bold are Paharia names and the purpose of making them in bold is to distinguish them from scientific names which are also in Italics and placed next to them*.

After compiling 193 IFs in the free listing activity, their taxonomic classification was attempted through a web-based literature search using their common names and photographs. Out of 193 foods, classification was available for 87 foods based on their common names in the Indian food composition tables and other related internet based databases (Table 3) while five food items were sent for identification after preparation of herbarium of their samples collected during their flowering and fruiting season (Figure 3).

### Commonly Consumed Indigenous Foods

Based on the FGDs, a short list of foods which were currently consumed by the community and were available for a significant duration in a year was prepared. This activity provided us with a total of 97 commonly consumed IFs out of 193 (50.3%). The taste, availability and accessibility of these foods were the common reasons cited by participants for routine consumption. Table 4 provides the list of commonly consumed IFs along with reasons for their consumption.

**Table 4:** Commonly consumed foods of Sauria Paharia community (*n* = 97).

**Food groups**	**Food items**	**Reasons for high consumption**			
		**Desirable taste**	**Easy to access**	**Abundant seasonal availability**	**Abundant perennial availability**
Cereals (*n =* 13)	Varieties of rice (***Swarna, Bhadai, Sarda, Sarap, Jesori, Bahyar, Bangla Bhat, Hariya, Banagal, Arwa, Lal Dhan***; *Oryza sativa* L.), Maize (***Makai***; *Zea mays* L.)	✓	✓		✓
	Pearl Millet (***Bajra/Shishua***; *Pennisetum typhoideum* Rich.)	✓	✓	✓	
Pulses (*n =* 5)	Cow pea, brown (***Jatta Usro***; *Vigna catjang* (L.) Walp.), Cow pea, white (***Eso Usro***; *Dolichos catjang* Burm.f), Rice bean (***Suthro***; *Phaseolus calcaratus* Roxb.), Horse gram, whole (***Kurthi***; *Dolichos biflorus* L.), ***Khesari*** (*Lathyrus sativus* L.)	✓	✓		✓
GLVs (*n =* 15)	Koinaar leaves (***Komo Ghasi***; *Bauhinia purpurea* L.), Amaranth leaves (***Adro Ghasi***; *Amaranth spinosus* L.), Bengal gram leaves (***Boot Ghasi***; *Cicer arietinum* L.), ***Khesari saag*** (*Lathyrus sativus* L.), Malabar spinach (***Pondka saag***; *Basella rubra* L.), Sinduar leaves (***Chilo/Kodgo Ghasi***; *Celosia argentia* L.), Aradiyo^*^, Mannadro^*^	✓	✓	✓	
	Potato leaves (***Aloo Ghasi***; *Solanum tuberosum* L.), Ponnaganni (***Gocchi Ghasi***; *Alternanthera sessilis* (L.) R.Br. ex DC.), Banyan leaves (***Pakkedi Ghasi***; *Ficus benghalensis* L.), ***Chiniya saag*** (*Brassica campestris* L.)		✓	✓	
	Drumstick leaves (***Sanjhori saag***; *Moringa oleifera* Lam.), Colocasia Leaves *(**Makedi Ghasi***; *Colocasia antiquorum* Schott), Bottle gourd leaves *(**Lol Ghasi***; *Lagenaria vulgaris* Ser.)		✓		✓
Other vegetables (*n =* 10)	Indigenous bitter gourd (***Bir Karela***; *Momordica charantia* L.), Indigenous field beans (***Simbi***; *Dolichos lablab* L.), Indigenous ridge gourd (***Jhingli***; *Luffa acutangula* (L.) Roxb.), Spine gourd (***Kokri***; *Momordica dioica* Roxb.), Kovai (***Kundri***; *Coccinia cordifolia* (L.) Cogn.), ***Pindra*** (*Flacourita indica* (Burm f.) Merr.), Kachnar flower (***Kachna Phool***; *Bauhinias variegata* L.), Ash gourd (***Zarkunda***; *Benincasa hispida* (Thunb.) Cogn.), Ziruli Flower (***Zaraael Phool***; *Indigofera cassioides* DC.), Drumstick Flower (***Sanjhori Phool***; *Moringa oleifera* Lam.)	✓	✓	✓	
Roots and tubers (*n =* 8)	Colocasia (***Makedi***; *Colocasia antiquorum* Schott), Ole (***Singla***; *Amorphophallus paeoniifolius* (Dennst.) Nicolson)	✓	✓		✓
	Yam Beans (***Misrikand*****;** *Pachyrhizus erosus* (L.) Urb.), Red Potato (***Lal Aloo***; *Solanum tuberosum* L.)		✓	✓	
	***Nappe*** (*Dioscorea pentaphylla* L.), Churke/Churka^*^, Alli^*^, Pangdro^*^	✓	✓	✓	
Fruits (*n =* 9)	Zizyphus (***Ber/Ilkarpu***; *Zizyphus jujube* Mill.), Palmyra fruit (***Tamli***; *Borassus flabellifer* L.), ***Piyaara*** (*Buchanania lanazan* Spr.), *Ambada* (***Ambad Pupu***; *Spondias mangifera* Wild.), Mahua, ripe (***Madgi***; *Madhuca indica* J.F. Gmel.), Tumki (***Telo/Kaanda/Kaande***; *Diospyros melanoxylon* Roxb.), Kusum Fruit (***Pusra***; *Scheleichera oleosa* (Lour.) Merr.), ***Dahu/Tisgo Chagzo*** (*Autocarpus lakoocha* Roxb.)	✓	✓	✓	
	***Dumari*** (*Ficus glomerata* Roxb.)		✓		✓
Flesh Foods (*n* = 24)	***Potta** (Burbus* spp.), Snail *(**Ghongri**; Pila globoasa)*, Mussels *(**Maako/Jhinuk;** Margaritifera margaritifera)*, Silhan Fish *(**Silong**; Silonia silondia)*, Freshwater Eel (***Gacchi***; *Anguilla Anguilla*), Banjakudi^*^, Field rat (***Moosaa***; *Rattus argentiventer*), Peacock (***Chuwa***; *Pavo cristatus*), Wild Pig (***Jangli suar/Kissu***; *Sus scrofa*), Indigenous Hen (***Jangli Murgi***; *Galloanserae*), Squirrel meat (***Gilhari ka mas***; *Scieuridea*), Patridge (***Teetar Chidiya**; Gray francolin*), Tirikado Chidiya^*^, Pura Bird^*^	✓	✓	✓	
	Catfish *(**Tonger/Tengra***; *Mystus vittatus*), Snake Head Fish (***Gadai/Gowari*****;** *Channa punctate*), ***Singhi** (Saccobranchus fossilis)*, Walking Catfish (**Mangri/Magur**; Clarias batrachus), Chatarkati^*^, Chachara^*^, Chuchi^*^, Gotti^*^, Chete^*^, Erke^*^,	✓	✓		✓
Mushrooms (*n =* 10)	Patangallo/Phutka^*^, Kero/Orho^*^, Telokuti/Telokuto^*^, Adro/edro/endro^*^, Jambuajo^*^, Maangro/Maako^*^, Takna^*^, Tero^*^, Chaariyoni^*^, Koro/Orho^*^	✓	✓	✓	
Insects for honey (*n* = 3)	Haubudu^*^, Teni^*^, Isge^*^	✓	✓		✓

*Text in italics and bold are Paharia names and the purpose of making them in bold is to distinguish them from scientific names which are also in Italics and placed next to them*.

* *Taxonomic classification not available*.

Among cereals, indigenous varieties of Rice (***Dhan***; *Oryza sativa*), Maize (***Makai****; Zea mays*) and Pearl millet (***Bajra****; Pennisetum typhoideum*) were commonly consumed in the study villages. Among rice varieties, most of the households reported growing ***Swarna***along with other indigenous varieties. Indigenous varieties such as ***Hariya, Bangla***& ***Bahiyar***were mostly found to be grown in hilly villages while ***Lal Dhan***, ***Arwa***& ***Jesori***were grown in villages situated along plains. Many indigenous and non-indigenous varieties of pulses were reported to be consumed throughout the year. Among GLVs, many varieties were hugely preferred for their taste, however most of these were available in specific seasons. Koinaar leaves (***Komo Ghasi***; *Bauhinia purpurea)*, an indigenous GLV, was reported to be popular for its flavorsome taste and satiety giving properties. As one FGD participant said:

“*Komo is liked but we do not get it often. If you make it once and eat, you won't feel hungry for the entire day. Won't even need rice.”* (Villager, Village Three, Study Block Two).

In contrast, Drumstick leaves (***Sanjhori saag****; Moringa oleifera*) was not liked for its taste but was consumed by many due to its abundant availability in open access areas in the village and at the *Baaris*.

Several indigenous varieties of vegetables and fruits were routinely accessed due to their taste and abundant seasonal availability. Most of the roots and tubers were preferred for their taste and commonly consumed throughout the year. ***Nappe***(*Dioscorea pentaphylla*), an indigenous tuber, was found to be popular for its taste and satiety properties. However, its consumption has declined with time due to its tedious pre-cooking processing. As a consequence, these indigenous roots have been largely replaced by non-indigenous varieties such as potatoes which are commonly accessed from markets or grown in the *Baaris*. One participant stated:

“*Earlier, Nappe was consumed a lot. It would be sliced big and then kept in the river (to remove toxins) and then would be steamed and eaten the next day. No need of a proper meal after that.”* (Villager, Village Two, Study Block Two).

Whereas, another participant said:

“*Potato is the one that is available all the time. We do not feel like eating it, but still eat it.”* (Villager, Village Three, Study Block One).

Many of the flesh foods were reportedly consumed throughout the year. Few of the indigenous fishes, such as, Freshwater Eel *(****Gacchi***; *Anguilla Anguilla)*, ***Banjakudi Machhli*** (taxonomic classification NA), Puti Machhli (***Pothi/Potha Machhli****; Burbus* spp.), Snails (***Ghongri***; *Pila globoasa*), Mussels (***Maako/Jhinuk***; *Margaritifera margaritifera*) and Silhan Fish *(****Silong***; *Silonia silondia*) were found to be seasonally available and chiefly consumed for their taste. Animal meats such as Field rat (***Moosa***; *Rattus argentiventer*), Peacock *(****Chuwa***; *Pavo cristatus*), Wild Pig *(****Jangli suar/Kissu***; *Sus scrofa*) and ***Tirikado*** ***Chidiya***(taxonomic classification NA) were reported to be consumed during hunting periods in the specific month of March/April every year. Villagers also reported harvesting and consuming the honey produced from indigenous species of bees namely, ***Haubudu, Teni, Isge***(taxonomic classifications NA). Despite the knowledge and availability of many indigenous mushrooms, only ten mushrooms were found to be commonly consumed due to their taste and easy accessibility from forests and markets.

### Preferred IFs Among the Community

**Figure 4:**
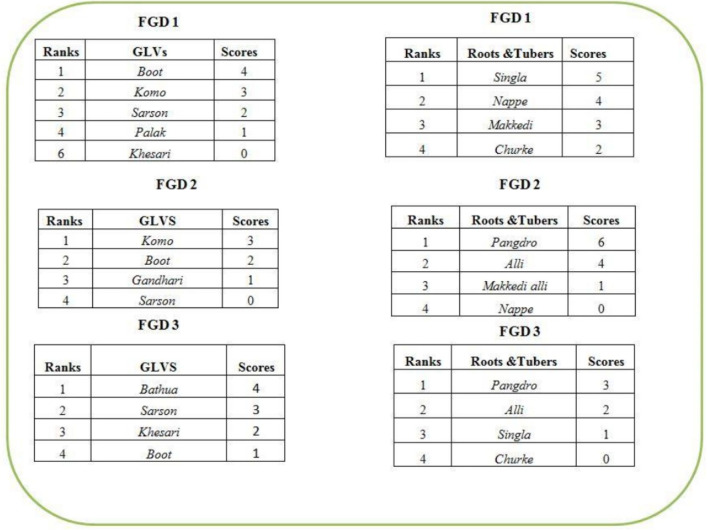
Pair wise ranking and scores to varieties of food items under different food groups.

The commonly consumed foods under each food group in a specific season were compared and ranked using pairwise ranking based on criterion like taste, availability, ease of production or collection by the community. These foods mainly comprised of several indigenous varieties although few non-indigenous foods were also found to be preferred over IFs. Figure 4 provides some examples of scoring and ranking of GLVs and roots & tubers.

Rice (***Dhan*)** and Maize (***Makai)***were the most preferred cereal food items. The community preferred indigenous varieties for their better taste, satiety and cooking properties. As one participant stated:

“*The indigenous one, is tasty and provides strength. If we consume hybrid, we digest it quickly and we feel hungry again*. *Hybrid variety gets digested very fast, therefore we feel hungry repeatedly. If we eat indigenous food, we feel full for a longer time duration. Indigenous food is good; hybrid food is lighter”*. (Villager, village Four, Study Block One).

Another participant said:

“*Indigenous is tastier. It cooks faster”. (*Villager, village Three, Study Block One).

This suggests that some of these indigenous varieties are more desirable in terms of taste and preparation convenience. However, the number of varieties had declined and the community was mostly sowing 2–3 varieties. They also consumed some hybrid varieties.

Non-indigenous red gram pulse (***Rehad*;** *Cajanus cajan*) was the most preferred variety of legume followed by indigenous varieties Cow pea, brown (***Barbatti***; *Vigna catjang*), Rice bean (***Suthro***; *Phaseolus calcaratus*) and Horse gram (***Kurthi***; *Dolichos biflorus*). Among indigenous GLVs, Bengal gram leaves (***Boot Ghasi***; *Cicer arietinum*) and Koinaar leaves (***Komo Ghasi***) had highest scores and were ranked as the most preferred varieties in all villages. Other GLV varieties that were preferred in multiple villages included Amaranth leaves (***Adro Ghasi****; Amaranth spinosus*), Drumstick leaves (***Sanjhori Saag***), Sinduar leaves (***Chilo/Kodgo Ghasi***; *Celosia argentia*), ***Khesari saag***(*Lathyrus sativus*), Colocasia Leaves *(****Makedi Ghasi***; *Colocasia antiquorum*), and ***Aradiyo Ghasi***(taxonomic classification NA). *Bathua* leaves (***Bathua***; *Chenopodium album*) and Mustard leaves (***Sarson***; *Brassica juncea*) were highly preferred non-indigenous GLVs due to their easy accessibility through markets.

Among indigenous vegetables, Field beans (***Simbi***; *Dolichos lablab*) and Spine gourd (***Kokri***; *Momordica dioica*) were the most liked varieties and received higher scores. In four of the study villages, indigenous Ridge gourd (***Jhingli***; *Luffa acutangula*), Kovai (***Kundri***; *Coccinia cordifolia*) and ***Pindra***(*Flacourita indica*) emerged out as commonly preferred vegetables while flowers of Kachnar (***Kachna Phool***; *Bauhinias variegata*) and Ziruli (***Zarael*** ***Phool****; Indigofera cassioides*) were reportedly preferred in only one study village. In case of roots and tubers, Ole (***Singla***; *Amorphophallus paeoniifolius*) received maximum score followed by ***Nappe***and ***Churke***(taxonomic classification NA). Taste preferences were seen for ***Alli***(taxonomic classification NA) and Colocasia (***Makedi*)** in multiple villages while ***Pangdro***(taxonomic classification NA) was found to be preferred in only one study village. Among fruits, Tumki (***Telo/Kaanda/Kaande***; *Diospyros melanoxylon*) got the highest score followed by ***Piyaara***(*Buchanania lanazan*), Zizyphus (***Ber/Ilkarpu***; *Zizyphus jujube*) and *D****ahu/Tisgo Chagzo***(*Autocarpus lakoocha*). Preference for non-indigenous varieties of Mango (***Aam***; *Mangifera indica*), Jackfruit (***Kathal***; *Artocarpus heterophyllus*) and Guava (***Amrood***; *Psidium guajava*) was seen in many villages. Among the indigenous varieties of commonly consumed mushrooms (*n* = 10), ***Telo-kuti***(taxonomic classification NA*)* was the most preferred mushroom, followed by ***Takna, Pattanglo*, **and ***Bandha-aero***(taxonomic classification NA).

### Little Used or Historically Consumed IFs Within the Community

Based on the information from FGDs, almost half (*n* = 96/193; 49.7%) of the IFs were found to be either little used or historically consumed. Common reasons cited by the participants included undesirable taste, long processing and cooking time and poor availability and accessibility. These foods along with the reasons for their non-consumption are listed in Table 5.

**Table 5:** List of little used or historically consumed IFs of Sauria Paharia community (*n* = 96).

**Food group**	**Food items**	**Reasons for low consumption**			
		**Undesirable taste**	**Difficult to access**	**Long cooking procedure**	**Limited availability**
Cereals (*n* = 3)	Sorghum (***Jowar*****, Desi**; *Sorghum vulgare* Pers.), Gondli^*^	✓	✓		✓
	*Ragi* [***Kodo/Mandua**; Eleusine coracana* (L.) Gaertn.]		✓		✓
Pulses (*n* = 1)	***Kusa*** [*Mucuna pruriens* (L.) DC.]		✓	✓	✓
GLVs (*n* = 21)	Kantha leaves *(**Daav Ghasi***; *Euphorbia granulate* Forssk.), Mata leaves [***Tisso Ghasi***; *Antidesma diandrum* (Roxb.)], Kena leaves *(**Berbayo Ghasi***; *Commelina benghalensis* L.), Garlic Leaves *(**Nasni Ghasi***; *Allium sativum* L.), Ziruli leaves (***Zaraael saag**; Indigofera cassioides* DC.), Gobero Adro^*^, Pusre Adro^*^, Gutni^*^, Chiroti Saag^*^, Acchadro^*^, Ursudi Ghasi^*^, Jonya Ghasi^*^, Kannasedi saag^*^, Bodo Ghasi^*^, Dababotri Ghasi^*^, Kotua saag^*^		✓		✓
	Dhurup leaves *(**Kondi Ghasi***; *Leucas lavandulifolia* Sm.), ***Naolo Ghasi*** (*Trianthema portulacastrum* L.), Sunsuni leaves *(**Junjuni***; *Marsilea minuta* L.), ***Sonpu saag*** (*Crotolaria juncea* L.), Madhari saag^*^	✓			
Other vegetables (*n* = 4)	***Sonpu Phool*** (*Crotolaria juncea* L.), ***Ber alli*** (*Dioscorea* spp.)		✓		✓
	Bamboo, tender *(**Maas Adro/Karu*****/*****Baans***; *Bambusa vulgaris* Schrad.), Barbatti Vegetable [***Jatta Usro***; *Vigna catjung* (L.) Walp.]	✓			
Roots and tubers (*n* = 15)	Pornialli^*^, Chalangan/Chalgo^*^, Chalko^*^, Chambiyalli^*^, Gumalli^*^, Isaha Alli^*^, Aeli^*^, Gomo^*^, Igzol^*^, Panne^*^, Keso^*^		✓	✓	
	***Ber alli** (Dioscorea* spp.), Taalko^*^	✓	✓		
	***Pindra*** [*Flacourita indica* (Burm f.) Merr.], Jattali^*^		✓		
Fruits (*n* = 10)	Marking Nut *(**Kero/Keero Toso***; *Semecarpus anacardium* L.f.), ***Haani*** (*Ficus exasperata* Vahl.), ***Tisso*** [*Antidesma diandrum* (Roxb.)], Banyan Fruit *(**Pakkedi***; *Ficus benghalensis* L.), Wood apple [***Bel/Otte***; *Aegle marmelos* (L.) Correa], Anni^*^Zara aeli^*^, Kaisge^*^, Kaita^*^, Dhela^*^		✓		✓
Flesh foods (*n* = 19)	***Chala/Chalgo*** (*Salmostoma acinaces*), Wallago *(**Boari***; *Wallago attu*), Eherchali^*^, Bele Fish *(**Baale**; **Glossogoboius giuris***); Mustura^*^, Doke^*^, Mitra^*^, Jambuchett^*^, Zimali^*^, Eggs of red ants ***(Chetado ka anda**; Aceophywlla smaragdina)*, Pigeon (***Pervan*****;** *Columba livia domestica*)		✓		✓
	Cat (***Mahalo/Mahala*****;** *Felis catus*), Porcupine **(*****Kissa/Chitru***; *Erethizon dorsatum*), Quail (***Bater***; *Coturnix coturnix*), Parrot (***Edru/Tota***; *Psittacine*), Tenga^*^, Kafo^*^, Tura^*^, Oda^*^		✓		
Mushrooms (*n* = 23)	Paarango^*^, Baado^*^, Naango^*^, Baansosu^*^, Baalco^*^, Ado^*^, Kerusudo^*^, Pattodi/Pittodi^*^, Chaandi^*^, Gobruosu^*^, Baanipoto^*^, Kuttapuda^*^, Bandho aero^*^, Bagdoto^*^, Jhinganu/Jhingan^*^, Naolo Osu^*^, Balo Osu^*^, Mokro/Moker osu^*^, Patla aero^*^, Isuno^*^, Jinpro aero^*^, Ganda Budi^*^, Kaijo^*^		✓		✓

*Text in italics and bold are Paharia names and the purpose of making them in bold is to distinguish them from scientific names which are also in Italics and placed next to them*.

* *Taxonomic classification not available*.

Few indigenous cereal varieties (3 out of 16) were found to be little consumed due to their unacceptable flavor and limited availability. The taste of Sorghum (***Jowar* desi**, *Sorghum vulgare*) was not appreciated and hence not consumed. Finger millet (***Kodo/Mandua***; *Eleusine coracana*) was reportedly liked for its taste but not routinely consumed due to its decreasing agricultural production over the years. Low access was reported for ***Kusa*** (*Mucuna pruriens*), an indigenous variety of pulse, due to its declining yield over the years and the long processing time like leaching the bean in running water for several hours before it is fit for consumption. A large number of GLVs (58.3%, *n* = 21) were found to be rarely consumed while four indigenous varieties of other vegetables were infrequently consumed. Among roots and tubers, many varieties (15 out of 23) were found to be little used due to the opportunity cost involved in accessing and processing these foods. Most of the indigenous fruits (52.6%, *n* = 10) were preferred for their taste but were difficult to access. Marking Nut *(****Kero/Keero Toso***; *Semecarpus anacardium*), a fruit, was found to be known for its medicinal properties and was mainly consumed by sick people*:*

“*We eat Kero sometimes when we are sick. We get better by eating Kero.”* (Villager, village one, Study Block One)

In case of flesh foods, around 44% (*n* = 19) foods were seen to be either little used or historically consumed. Meats from animals such as Porcupine (***Kissa/Chitru***; *Erethizon dorsatum*), Wild Cat (***Mahalo/Mahala***; *Felis catus*), Quail (**B*ater***; *Coturnix coturnix*), Parrot (***Edru/Tota***; *Psittacine*) ***Tenga, Kafo*,** ***Tura*, **and ***Oda***(classification NA) were reported to be rarely consumed by the community as these animals were hunted in historical times and are not currently available in the forest during the annual hunting foray by the community. Poor availability was observed for many indigenous fishes as they were available for only limited durations in a year. In addition, these fishes are difficult to access due to the diminishing water levels in local rivers, lakes and ponds. About 70% (*n* = 23) mushrooms were reported to be little used due to their limited availability in monsoon season.

### Indigenous Foods Routinely Accessed by the Community

Based on the agricultural diversity assessment tool and the market surveys, foods (indigenous as well as non-indigenous or hybrid) that were accessed in different seasons during the previous year by Sauria Paharia households were listed (Table 6). These foods were either cultivated in the agricultural lands, cleared forests (*Kurwa*), or kitchen gardens (*Baari*); collected from forests, open areas in the village or nearby water bodies; and bought from the local market.

**Table 6:** Home grown and local market foods (indigenous, non-indigenous, or hybrid) that were accessed in different seasons during the previous year by Sauria Paharia community.

**S. no. and food group**	**Local name/name of the variety**	**Food item (common name)**	**Place where produced/accessed/collected/purchased**	**(Indigenous/Non-indigenous/Hybrid)**	**Seasons (summer/monsoon/winter/all seasons)**
**Cereals**					
1	*Swarna Dhan*	Rice	Agricultural land/market	Indigenous	All seasons
2	*Lal Dhan*	Rice	Agricultural land	Indigenous	Summer
3	*Sarda Dhan*	Rice	Market	Indigenous	All seasons
4	*Arwa Dhan*	Rice	Market	Indigenous	Monsoon and/or Winter
5	*Sarap Dhan*	Rice	Agricultural land	Indigenous	Monsoon and/or Winter
6	*Jesori Dhan*	Rice	Agricultural land	Indigenous	Monsoon and/or Winter
7	*Bahyar Dhan*	Rice	Agricultural land	Indigenous	Monsoon and/or Winter
8	*Bangla Bhat*	Rice	Agricultural land	Indigenous	Monsoon and/or Winter
9	*Hariya Dhan*	Rice	Market	Indigenous	Summer
10	*Banagal Dhan*	Rice	Market	Indigenous	Summer
11	*Makai-Potio Gangi*	Maize	Agricultural land/Baari/market	Indigenous	All seasons
12	*Makai-Chamberi Gangi*	Maize	Kurwa	Indigenous	Summer
13	*Bajra/Shishua*	Pearl millet	Agricultural land/Kurwa	Indigenous	All seasons
14	*Gehun Ata*	Wheat flour	Market	Non-indigenous	All seasons
15	*Gehun*	Wheat	Agricultural land	Hybrid	Summer
16	*Dhan*	Rice	Agricultural land/Market/PDS	Hybrid	All seasons
17	*Makai*	Maize	Kurwa	Hybrid	All seasons
**Pulses**					
1	*Barbatti-Eso Usro*	Cowpea, white	Agricultural land/Kurwa/Baari/market	Indigenous	All seasons
2	*Barbatti- Jatta Usro*	Cowpea, brown	Agricultural land/Kurwa/Baari	Indigenous	All seasons
3	*Sutro Dal*	Rice bean	Agricultural land/Kurwa/Market	Indigenous	All seasons
4	*Kurthi Dal*	Horse gram	Agricultural land/Kurwa	Indigenous	All seasons
5	*Khesari Dal*	Khesari Dal	Market	Indigenous	Monsoon and/or Winter
6	*Chota Chana*	Bengal gram	Agricultural land/Market	Non-indigenous	All seasons
7	*Rehad Dal*	Red gram	Agricultural land/Kurwa/Baari/Market	Non-indigenous	All seasons
8	*Kurthi Dal*	Horse gram	Kurwa	Non-indigenous	Monsoon and/or Winter
9	*Masoor Dal*	Lentil	Market	Non-indigenous	Monsoon and/or Winter
10	*Soya bean*	Soya bean	Market	Non-indigenous	Monsoon and/or Winter
11	*Moong Dal*	Green gram	Market	Non-indigenous	All seasons
12	*Chana Dal*	Bengal gram	Agricultural land	Hybrid	Monsoon and/or Winter
13	*Barbatti Dal*	Cowpea, white	Kurwa	Hybrid	Monsoon and/or Winter
**Green leafy vegetables**					
1	*Adro Ghasi*	Amaranth leaves	Baari/Market	Indigenous	Summer
2	*Gobero Adro*		Forest	Indigenous	Summer
3	*Gocchi Ghasi*	Ponnaganni	Forest	Indigenous	Summer
4	*Pakkedi Ghasi*	Banyan leaves	Forest	Indigenous	Summer
5	*Komo Ghasi*	Koinaar leaves	Forest	Indigenous	Summer
6	*Manadro Saag*	-	Market	Indigenous	Summer
7	*Kodgo/Chilo Ghasi*	Sinduar leaves	Market	Indigenous	Summer
8	*Zaraael Ghasi*	Ziruli leaves	Forest	Indigenous	Monsoon and/or Winter
9	*Aradiyo Ghasi*	-	Forest	Indigenous	Monsoon and/or Winter
10	*Sanjhori Saag*	Drumstick leaves	Market	Indigenous	Monsoon and/or Winter
11	*Boot Ghasi*	Bengal gram leaves	Market	Indigenous	Monsoon and/or Winter
12	*Chiniya Saag*	-	Market	Indigenous	Monsoon and/or Winter
13	*Bandh Ghobi*	Cabbage leaves	Market	Non-indigenous	Summer
14	*Palak Saag*	Spinach leaves	Market	Non-indigenous	All seasons
15	*Mooli Saag*	Radish leaves	Market	Non-indigenous	Summer
16	*Sarson Saag*	Mustard leaves	Agricultural land/Kurwa/Baari/Market	Non-indigenous	All seasons
17	*Bathua Saag*	Bathua leaves	Market	Non-indigenous	Monsoon and/or Winter
18	*Pyaaz Saag*	Onion leaves	Market	Non-indigenous	Monsoon and/or Winter
19	*Dhaniya*	Coriander leaves	Market	Non-indigenous	Monsoon and/or winter
**Vegetables**					
1	*Kokri*	Spinegourd	Baari	Indigenous	Monsoon and/or winter
2	*Jhingli*	Ridgegourd	Baari	Indigenous	Monsoon and/or winter
3	*Bir Karela*	Bittergourd	Market	Indigenous	All seasons
4	*Kundri*	Kovai	Market	Indigenous	Monsoon and/or winter
5	*Kachna Phool*	Kachnar flower	Forest	Indigenous	Monsoon and/or Winter
6	*Zaraael Phool*	Ziruli flower	Forest	Indigenous	Monsoon and/or Winter
7	*Sem/Simbi*	Field beans	Market	Indigenous	Summer
8	*Sanjhori Phool*	Drumstick flower	Market	Indigenous	Summer
9	*Pindra*	-	Forest	Indigenous	Summer
10	*Kheera*	Cucumber	Agricultural land/Baari	Non-indigenous	All seasons
11	*Jhingli*	Ridgegourd	Market	Non-indigenous	Monsoon and/or Winter
12	*Hari mirch*	Green Chili	Baari/Market	Non-indigenous	Monsoon and/or Winter
13	*Baingan*	Brinjal	Baari/Market	Non-indigenous	All seasons
14	*Parwal*	Parwar	Market	Non-indigenous	Monsoon and/or Winter
15	*Kohda*	Pumpkin	Market	Non-indigenous	Monsoon and/or Winter
16	*Bhindi*	Ladyfinger	Market	Non-indigenous	All seasons
17	*Phool Ghobi*	Cauliflower	Market	Non-indigenous	All seasons
18	*Papita/Man Kunda*	Papaya, Raw	Market	Non-indigenous	Monsoon and/or winter
19	*Tamatar*	Tomato	Market	Non-indigenous	All seasons
20	*Kathal*	Jackfruit, Raw	Market/open space	Non-indigenous	Summer
21	*Kaccha Kela*	Banana, raw	Market	Non-indigenous	Summer
22	*Beans*	French beans	Market	Non-indigenous	Summer
**Roots and tubers**					
1	*Makedi*	Colocasia	Forest/market	Indigenous	Monsoon and/or winter
2	*Singla*	Ole	Market	Indigenous	Monsoon and/or winter
3	*Nappe*	–	Market	Indigenous	Monsoon and/or winter
4	*Lal Aloo*	Red potato	Market	Indigenous	Summer
5	*Aloo*	Potato	Agricultural land/Baari/Market	Non-indigenous	All seasons
6	*Pyaaz*	Onion	Baari/Market	Non-indigenous	All seasons
7	*Shakarkand*	Sweet Potato	Baari	Non-indigenous	Summer
8	*Lehsun*	Garlic	Market	Non-indigenous	All seasons
9	*Adrak*	Ginger	Market	Non-indigenous	All seasons
10	*Gajar*	Carrot	Market	Non-indigenous	All seasons
11	*Misrikand*	Yam beans	Market	Indigenous	Summer
**Fruits**					
1	*Madgi*	Mahua	Forest/open space	Indigenous	Summer
2	*Piyaara*	–	Forest	Indigenous	All seasons
3	*Telo/Kande/Kanda*	Tumki	Forest	Indigenous	Summer
4	*Imli*	Tamarind	Forest	Non-indigenous	Summer
5	*Jamun*	Blackberry	Forest	Non-indigenous	All seasons
6	*Aam*	Mango	Forest/open space	Non-indigenous	All seasons
7	*Khajur*	Dates	Forest/open space	Non-indigenous	Summer
8	*Papita/Man Kunda*	Papaya	Forest/open space	Non-indigenous	Summer
9	*Kathal*	Jackfruit	Forest/open space	Non-indigenous	Monsoon and/or winter
**Flesh Foods**					
1	*Potha Machhli*	Puti fish	Water bodies/market	Indigenous	All seasons
2	*Erke Machhli*	–	Water bodies	Indigenous	Summer
3	*Chete Machhli*	–	Water bodies	Indigenous	All seasons
4	*Gadai Machli*	Snake head fish	Water bodies	Indigenous	All seasons
5	*Chachara Machli*	–	Water bodies	Indigenous	Summer
6	*Banjakudi Machhli*	–	Water bodies	Indigenous	All seasons
7	*Chuchi Machhli*	–	Water bodies	Indigenous	Summer
8	*Gacchi Machhli*	Freshwater eel	Water bodies	Indigenous	All seasons
9	*Mangri/Magur Machhli*	Walking catfish	Water bodies	Indigenous	All seasons
10	*Ghongri*	Snail	Water bodies	Indigenous	Summer
11	*Jhinuk/Jhimali*	Mussel	Water bodies	Indigenous	Summer
12	*Gotti Machhli*	–	Water bodies	Indigenous	Summer
13	*Chalgo/Chala Fish*	Silver razor belly minnow	Water bodies	Indigenous	Summer
14	*Silong Machhli*	Silhan fish	Water bodies	Indigenous	All seasons
15	*Chatarkati Machhli*	–	Water bodies	Indigenous	Monsoon and/or winter
16	*Jangli suar*	Pork	Forest/Market	Indigenous	All seasons
17	*Jangli Murga*	Chicken	Forest/market	Indigenous	All seasons
18	*Teetar ka maas*	Partridge	Forest	Indigenous	All seasons
19	*Gilhari ka maas*	Squirrel meat	Forest	Indigenous	All seasons
20	*Pura Bird*	–	Forest	Indigenous	All seasons
21	*Chuwa*	Peacock	Forest	Indigenous	Monsoon and/or winter
22	*Moosa*	Field rat	Forest	indigenous	Monsoon and/or winter
23	*Tirikado Chidiya*	–	Forest	indigenous	Monsoon and/or winter
24	*Golden Machhli*	–	Water bodies	Non-indigenous	All seasons
25	*Kachua*	Turtle	Water bodies	Non-indigenous	Summer
26	*Kakro*	Crab	Water bodies	Non-indigenous	All seasons
27	*Rohu Machhli*	Rohu Fish	Water bodies/Market	Non-indigenous	All seasons
28	*Katla Machhli*	Catla Fish	Water bodies/Market	Non-indigenous	Monsoon and/or Winter
29	*Khargosh*	Rabbit meat	Forest	Non-indigenous	All seasons
30	*Suar*	Pork	Livestock	Non-indigenous	All seasons
31	*Desi Murga*	Chicken	Livestock	Non-indigenous	All seasons
32	*Jhingi/Iccha*	Prawns	Water bodies	Non-indigenous	All seasons
33	*Bakri/Khasi*	Goat meat	Livestock/market	Non-indigenous	All seasons
34	*Bhed*	Mutton	Livestock/market	Non-indigenous	All seasons
35	*Desi Anda*	Egg (Hen)	Market	Non-indigenous	All seasons
**Mushrooms**					
1	*Kero/Orho osu*	–	Forest	Indigenous	Monsoon and/or Winter
2	*Takna osu*	–	Forest	Indigenous	Monsoon and/or Winter
3	*Telokuti*	–	Forest	Indigenous	Monsoon and/or Winter
4	*Putka/Pattanglo*	–	Forest	Indigenous	Monsoon and/or Winter

*Text in italics represents Paharia name*.

**Figure 5:**
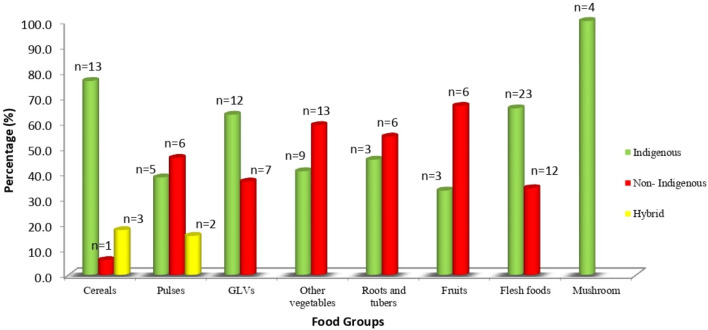
Distribution of foods accessed by Sauria Paharia community.

A total of 130 food items including both indigenous, non-indigenous and hybrid varieties were accessed by the study villages. Out of these, 56.9% (*n* = 74) were IFs. Though all these IFs were mentioned during the FGDs but this list is considerably lesser than the list of 193 IFs elicited during the free listing. Figure 5 shows the distribution of indigenous and non-indigenous or hybrid food varieties under different food groups that were routinely accessed. The community accessed several indigenous varieties of cereals, GLVs and flesh foods. Hybrid varieties were consumed only for cereals and pulses. The community accessed several non-indigenous varieties of vegetables which were either grown in their kitchen garden or bought from the market. Among indigenous varieties of roots and tubers accessed, some were purchased from the market while others were collected from forests or grown in the kitchen garden or agricultural lands. Very few indigenous varieties of fruits were accessed. These were mostly available in summer while a few were available perennially.

### Nutritive Value of IFs of Sauria Paharia Tribal Community

Based on the taxonomic classification, the nutritive value of 84 IFs were documented. Out of these, the nutritive values of 55 IFs were available from the food composition database for India (Indian Food Composition tables) (50) and other sources. The nutritive value of these foods are summarized in Supplementary Table 2. These 55 IFs comprised of 40 (72.7%) commonly consumed IFs while the rest were in little used or historically consumed category. A total of 29 food items were collected from the field and were sent for nutrient analysis to a NABL certified laboratory in Kolkata, India. Of these, majority (58.6%; *n* = 17) were commonly consumed while the rest were infrequently consumed. Table 7 provides the nutritive value of analyzed foods.

**Table 7:** Nutritive Value of Foods Analyzed in laboratory.

**S No**.	**Local names**	**Scientific name**	**Energy (Kcal/100 g)**	**Protein (g/100 g)**	**Carbohydrate (g/100 g)**	**Fat (g/100 g)**	**Dietary fiber (g/100 g)**	**B-Carotene (μg/100 g)**	**Vit C (mg/100 g)**	**Vit B1 (mg/100 g)**	**VitB2 (mg/100 g)**	**Iron (mg/100 g)**	**Zinc (mg/100 g)**	**Calcium (mg/100 g)**	**Folic acid (μg/100 g)**	**Phosphorus (mg/100 g)**
1	*Kusa*	*Mucuna pruriens* (L.) DC.	193	18.3	29.8	ND	14.46	22.42	3.01	ND	ND	5.32	1.8	945.38	ND	36.5
2	*Berbayo Ghasi*	*Commelina benghalensis* L.	27	2.5	4.11	ND	3.6	6741.75	5.97	ND	ND	42.9	0.77	130.83	1.1	48.02
3	Aradiyo Ghasi*		45	5.8	5.48	ND	4.21	942.69	6.04	0.64	0.74	77.66	1.22	231.76	ND	25.63
4	*Chilo Ghasi*	*Celosia argentia* L.	25	2.4	3.65	ND	2.51	4797.29	2.99	0.3	ND	7.87	0.59	150.53	ND	40.74
5	*Komo Ghasi*	*Bauhinia purpurea* L.	85	8.7	12.52	ND	4.22	2935.18	2.56	15.34	0.71	4.30	1.38	146.53	ND	ND
6	*Naolo Ghasi*	*Trianthema portulacastrum* L.	26	2.5	3.97	ND	1.45	5200.27	1.28	22.47	16.63	35.51	0.61	71.47	ND	ND
7	*Junjuni*	*Marsilea minuta* L.	113	7.4	20.76	ND	7.24	15332.71	1.28	2.3	2.6	16.56	3.5	90.118	ND	ND
8	Mannadro*		102	10.7	14.59	ND	13.5	316.38	2.95	ND	0.84	5.15	2.46	73.34	ND	63.35
9	*Pondka saag*	*Basella rubra* L.	21	3.1	2.03	ND	1.63	62.23	2.98	ND	ND	3.40	0.39	160.51	ND	25.26
10	*Aloo Ghasi*	*Solanum tuberosum* L.	38	6.2	3.22	ND	1.23	19850.51	3.57	0.5	ND	7.23	0.37	127.94	ND	0.023
11	*Khesari saag*	*Lathyrus sativus* L.	46	6.2	5.2	ND	2.27	5452.23	1.24	1.9	0.46	6.55	0.44	69.51	ND	0.023
12	*Tisso Ghasi*	*Antidesma diandrum* (Roxb.)	109	4.7	22.38	ND	7.63	2871.23	1.28	13.87	2.62	5.27	0.651	474.29	ND	ND
13	*Pindra/Pinra*	*Flacourita indica* (Burm f.) Merr.	73	4.5	13.78	ND	8.76	10.74	6.16	0.49	1.04	2.089	0.29	22.48	ND	33.23
14	*Ber alli* (Vegetable)	*Dioscorea* spp.	112	2.9	25.08	ND	8.09	ND	1.82	ND	ND	2.01	0.29	32.65	ND	398.36
15	*Kachna Phool*	*Bauhinias variegata* L.	83	2.9	17.7	ND	8.49	416.05	2.48	ND	0.3	3.44	0.64	404.91	ND	447.68
16	*Sonpu Phool*	*Crotolaria juncea* L.	120	2.9	27.04	ND	7.43	1112.72	1.77	3.09	ND	7.6	0.15	320.21	ND	537.27
17	*Sanjhori Phool*	*Moringa oleifera* Lam.	71	4.6	13.03	ND	5.23	260.86	2.43	0.48	0.57	5.24	0.72	80.53	ND	0.023
18	*Ber alli* (Root)	*Dioscorea* spp.	124	3.3	27.75	ND	6.96	ND	1.77	ND	ND	3.56	0.74	38.46	ND	366.34
19	Alli*		195	3.7	44.92	ND	12.29	ND	1.24	14.98	ND	7.03	0.5	43.4	ND	0.04
20	Chalangan/*Chalango		133	3.6	29.57	0.02	9.45	ND	1.21	1.05	0.86	9.53	1.17	151.5	ND	0.04
21	Pangdro*		61	3.1	12.26	ND	4.66	ND	1.23	1.2	0.26	1.17	0.49	16.86	ND	0.02
22	Churka/Churke*		42	6.7	3.77	ND	2.45	ND	3.02	ND	ND	50.55	0.53	42.64	ND	15.41
23	*Singla*	*Amorphophallus paeoniifolius* (Dennst.) Nicolson	64	6.3	9.65	ND	1.15	ND	3.05	ND	ND	11.1	1.08	35.71	ND	45.05
24	*Nappe*	*Dioscorea pentaphylla* L.	72	4.4	13.49	ND	1.34	ND	3.08	1.09	ND	55.87	0.63	33.22	ND	26.47
25	*Haani*	*Ficus exasperata* Vahl.	60	3.0	11.85	ND	9.27	ND	2.07	0.29	ND	6.56	0.44	275.16	ND	154.86
26	*Dahu*	*Autocarpus lakoocha* Roxb.	121	2.8	27.33	ND	7.2	1843.48	8.93	0.32	1.32	1.81	0.131	54.72	ND	ND
27	*Dumari*	*Ficus glomerata* Roxb.	52	3.2	9.7	ND	5.82	ND	ND	ND	ND	1.52	0.43	84	1.48	10.38
28	*Pusra*	*Scheleichera oleosa* (Lour.) Merr.	144	6.3	29.72	ND	14.89	6237.89	3.08	ND	ND	44.23	0.79	134.86	ND	51.3
29	Anni*		57	3.1	10.76	0.13	6.25	8.9	ND	ND	0.18	0.4	0.1	3.62	ND	9.99

*Text in Italics represents Paharia name*.

^*^Herbariums were prepared but could not be identified.

*ND, not detected*.

Among IFs for which nutritive values were available in secondary literature, the indigenous cereal, Finger millet, was specifically found to have high levels of thiamine (0.37 mg/100 g), calcium (364 mg/100 g) and dietary fiber (11.18 g/100 g). Many indigenous pulses namely Cow pea, brown (***Jatta Usro***; *Vigna catjang*), Cow pea, white (***Eso Usro***; *Dolichos catjang*), Rice bean (***Suthro***), Horse gram, whole (***Kurthi***), and ***Khesari Dal***(*Lathyrus sativus*) were rich sources of protein (range 21.5 to 28.2 g/100 g) and iron (range 5.04–8.76 mg/100 g). The folate levels of Horse gram and Cowpea, white, were high and varied from 163–249 μg/100 g. Most of the indigenous GLVs like Kantha leaves *(****Daav Ghasi***; *Euphorbia granulate)*, Drumstick leaves (***Sanjhor*i** ***Saag***) and Dhurup leaves *(****Kondi Ghasi***; *Leucas lavandulifolia*) were seen to have high levels of vitamin A (range 11, 680–18, 460 μg/100 g) and calcium (range 236–425 mg/100 g). The vitamin C content of GLVs like Colocasia Leaves (***Makedi Ghasi***), Ponnaganni (***Gocchi Ghasi***; *Alternanthera sessilis*) and Drumstick leaves was found to be high (range 40.7–108 mg/100 g). Colocasia leaves, in particular, was found to have high folate content (159 μg/100 g). Dhurup leaves, Bengal gram leaves (***Boot Ghasi***) and Kantha leaves had good levels of iron (range 20–81.09 mg/100 g). The vegetables consumed i.e., Kovai *(****Kundri***), and Bitter gourd (***Bir Karela***; *Momordica charantia*) were found to be rich sources of vitamin C (range 21.08 to 50.87 mg/100 g). The Zizyphus fruit (***Ber/Ilkarpu***) was found to be rich in vitamin C (60.93 mg/100 g). The fruit, Marking nut (***Kero/Keero Toso***) was found to have remarkably high levels of protein (26.4 g/100 g) and fairly good levels of iron (6.1 mg/100 g) and calcium (295 mg/100 g) respectively. Indigenous flesh foods apart from being rich sources of good quality proteins, were also rich in several micronutrients. High folate levels (1, 294 and 2, 438 μg/100 g) were seen for Silhan *(****Silong***) and Freshwater Eel. Eggs of red ants (***Chetado ka anda***; *Aceophywlla smaragdina*) had high levels of calcium (104 mg/100 g).

Out of the foods analyzed in the laboratory, several indigenous GLVs like ***Berbayo Ghasi*** (*Commelina benghalensis*), Koinaar leaves (***Komo Ghasi***), Khesari leaves (***Khesari*** ***saag***), Sunsuni leaves (***Junjuni***; *Marsilea minuta*), and Potato leaves (***Aloo Ghasi***; *Solanum tuberosum*) were found to be rich sources of vitamin A (range 2, 935–19, 850 μg/100 g). The GLV, ***Aradiyo Ghasi***was found to be exceptionally rich in iron (77.6 mg/100 g) and calcium (231.8 mg/100 g) while Sunsuni leaves had good levels of zinc (3.5 mg/100 g). Few of the GLVs like Koinaar leaves (***Komo***), Mata leaves (***Tisso*** ***Ghasi***; *Antidesma diandrum*), and ***Naolo Ghasi***(*Trianthema portulacastrum*) were found to have high thiamine content (range 13.87–22.47 mg/100 g). High riboflavin content of 16.6 mg/100 g was observed in ***Naolo Ghasi***. Fruits like ***Pusra***and ***Dahu***were seen to have high levels of vitamin A (6, 237 and 1, 843 μg/100 g). ***Dahu***, a fruit, had reasonable amount of Vitamin C (8.93 mg/100 g). The indigenous roots, ***Churka***and ***Nappe***were found to have very high iron content (50.55 and 55.87 mg/100 g). ***Alli***, was rich in dietary fiber (12.29 g/100 g) and thiamine (14.98 mg/100 g). The indigenous vegetable, ***Sonpu Phool***(*Crotolaria juncea*) was found to be rich in vitamin A (1, 113 μg/100 g) while Kachnar flower (***Kachna Phool***) had high calcium content (404.9 mg/100 g). The indigenous pulse ***Kusa***was found to be a rich source of protein (18.3/100 g), dietary fiber (14.46/100 g) and calcium (945.38 mg/100 g).

Some of the calcium rich IFs were found to have high phosphorus content such as Drumstick leaves (phosphorous content: 109 mg), Kachnar flower (phosphorous content: 447.7 mg), and Eggs of red ants (phosphorus content: 107 mg). However, all these foods owing to high calcium levels have ideal Calcium to Phosphorous ratio of around 2:1 which contributes to bone health.

**Figure 6:**
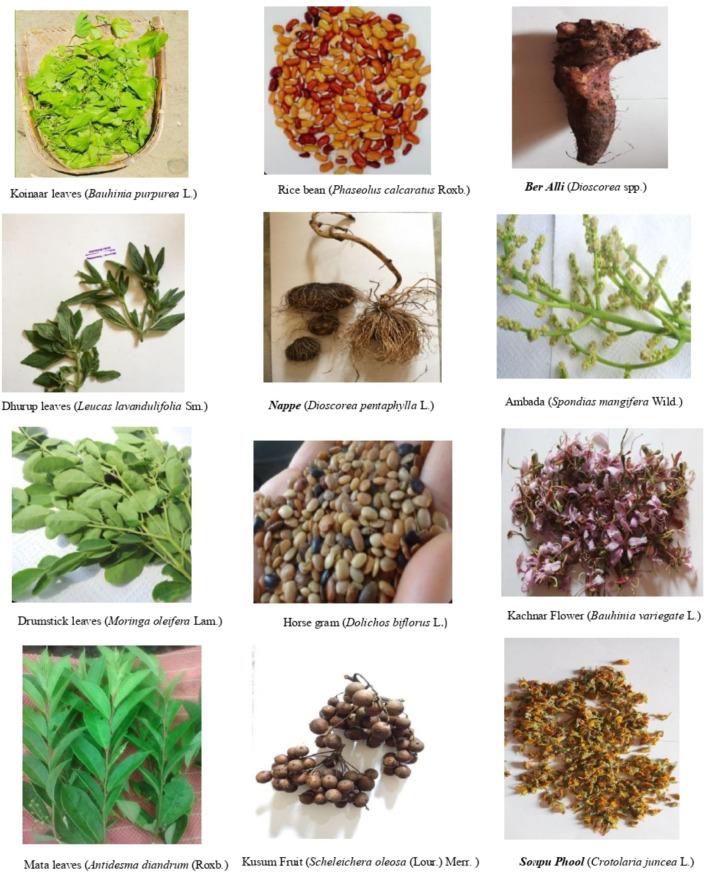
Some micronutrient rich indigenous foods of Sauria Paharia community. *Text in italics and bold are Paharia names and the purpose of making them in bold is to distinguish them from scientific names which are also in Italics and placed next to them*.

Based on these findings, we shortlisted 67 IFs that were found to be rich in various micronutrients. Most of these foods comprised of GLVs (29.9%) and flesh foods (20.9%). Roots and tubers along with other vegetables also contributed to more than 20% of the total micronutrient rich IFs. Few varieties of fruits (10.4%), pulses (9%) and cereals (6%) were seen to have high content of micronutrients. Majority of these micronutrient rich IFs were frequently consumed and accessed (65.7%) while the rest were used infrequently (Table 8). The pictures of some of these nutrient rich IFs are given in Figure 6.

**Table 8:** Micronutrient rich foods of Sauria Paharia community, Jharkhand (*n* = 67).

**Commonly consumed foods (*****n*** **=** **44)**		**Little used foods (*****n*** **=** **23)**	
**Food groups**	**Food items**	**Food groups**	**Food items**
Cereals	1. Maize (*Makai*) 2. Pearl millet (*Bajra/Shishua*)	Cereals	1.Finger millet (*Kodo/Mandua*) 2. Sorghum (*Jowar*)
Pulses	1.Cowpea, brown (*Jatta Usro*) 2. Cowpea, white (*Eso Usro)* 3. Horse gram *(Kurthi*) 4. *Khesari Dal* 5. Rice bean (*Suthro*)	Pulses	1. *Kusa*
GLVs	1.Koinaar leaves (*Komo Ghasi*) 2. Drumstick leaves (*Sanjhori saag*) 3. Amaranth leaves (*Adro Ghasi*) 4. Ponnaganni *(Gocchi Ghasi*) 5. Sinduar leaves *(Chilo/Kodgo Ghasi*) 6. Bengal gram leaves (*Boot Ghasi*) 7. Banyan leaves (*Pakkedi Ghasi*) 8. Malabar spinach (*Pondka saag*) 9. Potato leaves (*Aloo Ghasi*) 10. Chiniya saag^***^ 11. *Khesari saag* 12. Aradiyo Ghasi^***^ 13.Mannadro^***^	GLVs	1.Kantha leaves (*Daav Ghasi*) 2. Kena leaves (*Berbayo Ghasi*) 3. Dhurup leaves (*Kondi Ghasi*) 4. *Naolo Ghasi* 5. Sunsuni leaves (*Junjuni)* 6. Garlic leaves (*Nasni Ghasi*) 7. Mata leaves *(Tisso Ghasi*)
Other vegetables	1.Indigenous bitter gourd *(Bir Karela*) 2. Field beans, tender (*Simbi*) 3. Kachnar flower (*Kachnar* Phool) 4. Kovai (*Kundri*) 5. Drumstick flower (*Sanjhori Phool*) 6. Pindra^***^	Other vegetables	1. Sonpu Phool ^***^ 2. *Ber alli* 3. Cowpea, vegetable (*Jatta Usro*)
Roots and tubers	1.Colocasia (*Makedi)* 2. Ole (*Singla)* 3. *Nappe* 4. Churke/Churka^***^ 5.Alli^***^	Roots and tubers	1.*Ber alli* 2.Chalangan/Chalango^***^
Fruits	1.Zizyphus (*Ber/Ilkrapu*) 2. *Ambada* (*Ambad pupu*) 3. *Dahu* 4. Kusum fruit (*Pusra*)	Fruits	1.Haani ^***^ 2. Marking nut (*Kero/Keero/Toso)* 3. Banyan fruit (*Pakkedi)*
Flesh foods	1. Puti (*Potha Machli)* 2. Walking Catfish (*Mangri/Magur Machhli)* 3. *Singhi* 4. Freshwater Eel (*Gacchi Machhli*) 5. Catfish (*Tonger/Tengra Machhli*) 6. Snail (*Ghongri*) 7. Silhan Fish (*Silong Machhli*) 8. Mussels (*Maako/jhinuk*) 9. Pig (*Jangli suar/kissu*)	Flesh foods	1.Wallago (*Boari Machhli*) 2. Bele fish (*Baale Machhli*) 3. Eggs of red ants (*Chetado ka ande*) 4. Pigeon *(Pervan*) 5. Quail (*Bater*)

*Text in Italics represents Paharia name*.

*** *Taxonomic classification not available*.

## Discussion

Sauria Paharia tribes of Jharkhand displayed a rich traditional ecological knowledge about their indigenous foods and reported about 193 IFs. These included varieties of rice, GLVs, pulses, fruits, vegetables, roots and tubers mushrooms, insects, wild animal, and fishes. However, only about half (50%; *n* = 97) of these IFs were currently consumed by the community due to easy accessibility, availability and desirable taste. Based on the taxonomic classification nutritive value of 84 IFs were compiled. Among these, we found that 44 IFs that were routinely accessed were rich sources of protein, iron, calcium, vitamin A and Vitamin C. Interestingly, an additional 23 IFs that were little used for various reasons by the community were found to be nutrient rich.

The dietary staples of Sauria Paharias were predominantly indigenous varieties of rice. Though not many such indigenous varieties were sown and consumed by each household, a clear preference for and consistent practice of cultivation and consumption of indigenous rice was observed. The number of such indigenous rice varieties was 11. A grassroots organization *Pradan* in the region has compiled a list of 143 varieties of indigenous rice that were historically grown by this tribal group. However, the current practice has been reduced to 10 varieties (32). Though in limited numbers, another study has also confirmed the reliance on indigenous varieties of rice by this community. The common reasons cited in that study for this continued usage included a good demand for indigenous varieties in local market, a source of supporting livelihood of the community and the desirable characteristics of the varieties like drought and pest resistance (35). Some studies have also documented higher nutritional value of indigenous rice varieties consumed in the region (54–56). Some of the other probable reasons for Sauria Paharias, who are usually small and marginal farmers to continue growing indigenous varieties could be their geographic remoteness and dependability on low yet stable outputs of indigenous varieties especially in their rain fed agricultural and forest lands (57). The practice of growing indigenous rice among the Sauria Paharias is contrary to the trend of cultivation of hybrid and high yield varieties of rice by other tribals in the districts of Jharkhand and the overall pattern in Jharkhand which shows that <20% of the total production of rice is contributed by traditional indigenous varieties (58). Based on the above findings, this preference of Sauria Paharias toward cultivation of indigenous rice has a strong sustainability component which can also enhance the nutritional status of the community, provided the community is supported and empowered with knowledge and technology to support this traditional practice of production with enhanced and assured yield and higher consumption of the same. This would also be critical for the propagation of hybrid varieties in use today, whose sustainability itself may suffer if indigenous varieties stop being cultivated and are lost from the repertoire of rice varieties (58).

The other cereals consumed by the community included indigenous varieties of Pearl millet (***Bajra/Shishua***) and Maize (***Gangai***). Indigenous Finger millet (***Mandua/Kodo***), Sorghum (***Jowar***) and ***Gondli***were mentioned to have been historically grown in the region but the practice is currently discontinued. Similar consumption patterns of indigenous coarse cereals like maize namely ***Satia Makai****and* ***Ikhjo Gangai***; pearl millet like ***Sarve bajra****and* ***Moto bajra***have been documented in another study (35). However, the study by the grassroots NGO “*Pradan*” clearly highlights the diminishing basket of coarse cereals in the region which historically constituted of little millet, foxtail millet, and barnyard millet which the community has ceased to cultivate (32).

Indigenous varieties of pulses were grown in *Kurwas* and agricultural lands; several varieties of indigenous GLVs were grown and collected from forests or from agricultural lands as weeds; indigenous varieties of roots and tubers, mushrooms, and flesh foods were also reported. Other studies have also documented availability of several indigenous species of different food groups in the region (32). However, the free list compiled in our study has higher numbers of several species systematically listed with their taxonomic classification and nutritive values. To our knowledge there has been no systematic listing and documentation of IF resources of Sauria Paharia community of Jharkhand.

The current meal composition of Sauria Paharias consisting of rice with either a tuber (mostly potato) or GLVs or sometimes pulses or flesh foods, is similar to that observed in other studies on the same community as well as other tribal groups in the region (8, 32, 59). Though this community was non-vegetarian, their reliance on flesh foods that are either reared, hunted from the nearby forest or purchased from the market was comparatively lower. Another study documents a similar meal pattern in the tribal communities in the region at present. However, that study also underscores the historical dietary patterns recollected by village elders as hearty meals comprising of rice, dal, maize, maize bread, millet bread, millet porridge, vegetables procured from the forests and fields and fishes from nearby streams, all consumed in one single day (32).Despite a diverse list of indigenous food items, only 50% of the IFs were reported to be commonly consumed. Reasons for this could be a high opportunity cost of accessing some of the foods in terms of amount of time and energy required to forage them as well as an erosion in the traditional indigenous knowledge and usage of IFs that has been noticed among other indigenous communities in the region as well (8, 9, 59, 60). Some common reasons suggested for this decline include, migration of adults and youth to cities resulting in more reliance on market and public distribution system for grains and other foods; a changing agrarian practice of mono-cropping with diminished cultivation of indigenous cereals resulting in loss of indigenous seeds; promotion of high yield varieties of paddy due to urban influences; and dwindling natural resources resulting in reduced use of forest resources and water bodies for food (32).

Although there was an incongruity between the consumption of IFs and TEK about them, the pairwise ranking exercise demonstrated a strong preference toward IFs and their current consumption. The community also reported certain IFs that are not in use currently or little used but are valued for their taste. Seasonality and opportunity cost of collecting them from forest and the time required to process them before cooking were identified as reasons for their lower consumption. Additionally, the community also chronicled certain IFs with poor palatability but that, owing to their abundant availability were consumed during periods of scarcity. Other studies have also highlighted sensory acceptability, opportunity cost for accessing and preparing IFs, suboptimal agricultural productivity, abundant availability during periods of scarcity, and lack of awareness about IFs among present generation as factors associated with consumption pattern of IFs (61–63).

Many of the commonly consumed IFs were found to have high levels of micronutrients like iron, calcium, vitamin A, vitamin C and were rich sources of proteins. We also found several IFs from plant sources with high levels of Calcium (Ca) and Phosphorus (P), and their Ca/P ratio of around 2:1 was ideal for bone health. This is crucial as the community did not consume milk in their routine diets, and IFs rich in Calcium and Phosphorus could be a good substitute of milk which is desirable for bone health, provided they are adequately consumed (64, 65). Several studies from Asia, Africa and South America have demonstrated high nutritive value of wild plants and traditional indigenous foods and their consumption leading to better intakes of vitamins and minerals (66–69). Thus, nutrient rich IFs in the Sauria Paharia community could be effectively utilized and strategies to promote optimal consumption of these foods through nutrition education and counseling could positively impact nutritional intake in the community. In fact, a study in a tribal community of Telangana, India has demonstrated that nutrition security can be achieved by utilizing traditional farming systems. Thus, such interventions may prove to be a better option over bio fortification which is restricted in improving intake of limited nutrients and may also promote mono diets (70, 71).

Many of the little used IFs owing to their seasonality and accessibility were also found to be nutrient rich and some of them were also identified as highly palatable. Transposing the cultivation of such difficult to access yet highly palatable and nutritious wild species from the forest into the backyard gardens to meet the nutritional requirements of the communities is an interesting strategy that has been suggested in one study (72).

Many of the commonly consumed IFs especially GLVs and roots and tubers were weeds and did not require any cultivation. Interestingly, globally, close to 90% of the most widespread and flourishing weeds are edible, and many of these species have a high nutritional value and medicinal properties (73–77). Documentation and propagation of the TEK about the dietary utility of such weeds substantiated with scientific evidence on their palatability, safety and nutritive value can be a sustainable strategy of utilizing indigenous knowledge for re-shaping the food environment of this tribal community. FAO is coordinating efforts for the compilation of IF composition values from the seven socio-economic indigenous regions across the globe with Meghalaya, a North eastern state of India being one of them (78). Such a comprehensive compendium of region specific IFs to ensure the propagation, promotion and sometimes re-introduction of these foods into the daily diets of the indigenous communities could help to improve their intakes and address specific nutritional deficiencies (79).

## Limitations

- Though the study analyzed and compiled nutritive value of 84 IFs of Sauria Paharia community, owing to limitations around seasonal availability and accessibility, taxonomic classification could not be performed for all routinely consumed as well as little used IFs and as a result their nutritive value could not be explored.
- Though several foods were found to be rich sources of nutrients, it is important to assess the bioavailability of nutrients from these foods.

## Conclusion

With The United Nations Decade of Action on Nutrition (2016–2025) aiming to eradicate hunger and prevent all forms of malnutrition worldwide through focused policies and programmatic options for shaping food systems, the documenting, reviving, and propagating the IF systems of tribal communities of India may be one of the sustainable solutions. The present work on IFs of Sauria Paharia tribes highlights the fact that issues of nutrition security in this tribal community could be addressed by according value and prestige to this treasure trove of knowledge about IFs and its holders and promoting their consumption. A coherent policy for protection and revival of IF systems and utilization of the specific nutrient rich IFs of tribal communities as part of their daily diets while mainstreaming them into the dietary diversification strategies and ongoing supplementary feeding programs can go a long way in addressing nutritional well-being of the tribal communities of India. More broadly, it can help contribute to addressing SDG 2 and beyond. Fostering these sustainable food systems, and the rich biodiversity within them through supportive practices, has the potential to simultaneously improve the diets and nutrition of vulnerable populations while also increasing resilience of the communities in which they live.

## Data Availability Statement

All datasets generated for this study are included in the article/Supplementary Material.

## Ethics Statement

All procedures involving humans in this study were approved by the Institutional Ethics Committee at Indian Institute of Public Health-Delhi, Public Health Foundation of India and All India Institute of Medical Sciences, New Delhi. Administrative approvals from authorities at district level was also taken. Written informed consent was obtained from all participants who were literate. Third-party witnessed verbal consents were obtained from illiterate participants.

## Author Contributions

SG-J and AS conceived and designed the study with overall supervision from JF. SG-J, RK, and AS supervised the entire data collection process. RK and SB did the data analysis. SG-J, RK, SB, and AS prepared the first draft of the manuscript. SD critiqued and modified the draft. All authors contributed to critique and modification of the manuscript, read, and approved the final version. SG-J had final responsibility for the decision to submit for publication.

## Conflict of Interest

The authors declare that the research was conducted in the absence of any commercial or financial relationships that could be construed as a potential conflict of interest.

## Footnotes

^1^Species of plant/animal foods native to the place that are accessed through natural environment and are culturally acceptable (23)

^2^Species of plant/animal foods that are not native to the place and have been introduced through human activity, either deliberately or accidentally (46)

^3^Species of plants/animals that are created through cross-combination of two different species (47)

^4^Local names have been written in bold to differentiate from scientific names in results section.
