# Supplementary material for: Leveraging Traditional Ecological Knowledge and Access to Nutrient-Rich Indigenous Foods to Help Achieve SDG 2: An Analysis of the Indigenous Foods of Sauria Paharias, a Vulnerable Tribal Community in Jharkhand, India
Source: Front Nutr. 2020 Jun 2;7:61. doi: 10.3389/fnut.2020.00061 (PMC7280452; doi:10.3389/fnut.2020.00061)
Supplement: Supplementary file 1 [file Table_1.DOCX]

***Supplementary Material***

| **S. No.** | **Test Parameter/Standard** | **Methodology** | **Reference method** | **Limit of Quantification (LOQ)** |
| --- | --- | --- | --- | --- |
| 1. | Energy (Kcal/100gm) | Gravimetric method | IS:9487-1980(RA-2005) | 1.0 |
| 2. | Protein (g/100gm) | Titrimetric method | FSSAI Lab Manual | 0.1 |
| 3. | Total Fat (g/100gm) | Gravimetric method | IS:9487-1980(RA-2005) | 0.02 |
| 4. | Total Carbohydrate (g/100gm) | By difference | IS:1656-2007 | 0.1 |
| 5. | Dietary Fibre (g/100gm) | Enzymatic Gravimetric method | AOAC 20^th^ edition 985.29 | 0.5 |
| 6. | Vitamin A (as β- carotene) (µg/100gm) | High Performance Liquid Chromatography (HPLC) | QA.16.5.163 | 5.0 |
| 7. | Vitamin B_1_,B_2_( mg/100gm) | High Performance Liquid Chromatography (HPLC) | QA.16.5.9 | 0.2 |
| 8. | Vitamin C (mg/100gm | High Performance Liquid Chromatography (HPLC) | IS:5838-1970 (RA-2005) | 1.0 |
| 9. | Folic acid (µg /100gm) | High Performance Liquid Chromatography (HPLC) | QA.16.5.9 | 0.1 |
| 10. | Calcium, Iron, Zinc (mg/100gm) | Inductively Coupled Plasma Mass Spectrometry (ICP-MS) | QA.16.5.2/AOAC 20^th^ edition | 0.05 |

**Supplementary Table 1: List of parameters & relevant methodological details for nutrient analysis**

**Supplementary Table 2: List of food items with their nutritive value available from the Indian food composition tables and other sources**

| **S. No.** | **Food items** | **Common name (English/**  **Hindi)** | **Scientific name** | **Energy (Kcal/**  **100g)** | **Protein  (g/**  **100g)** | **Carbohydrate (g/ 100g)** | **Fat (g/ 100g)** | **Dietary fibre (g/ 100g)** | **β- Carotene/**  **Retinol (µg/ 100g)** | **Vit C**  **(mg/ 100g)** | **Vit B1 (mg/ 100g)** | **Vit B2 (mg/ 100g)** | **Iron (mg/ 100g)** | **Zinc (mg/ 100g)** | **Calcium (mg/ 100g)** | **Folic acid (µg/ 100g)** | **Phosphorus (mg/ 100gm)** |
| --- | --- | --- | --- | --- | --- | --- | --- | --- | --- | --- | --- | --- | --- | --- | --- | --- | --- |
|  | Rice, (indigenous)* | Rice, raw, milled | *Oryza sativa* L. | 343 | 7.94 | 78.24 | 0.52 | 2.81 | 28.23 | NA | 0.05 | 0.05 | 0.65 | 1.21 | 7.49 | 9.32 | 96 |
|  | *Makai/Gangi** | Maize | *Zea mays* L. | 120 | 3.6 | 22.69 | 1.4 | 3.67 | 36.27 | NA | 0.17 | 0.12 | 0.71 | 0.97 | 6.35 | 62.96 | 163 |
|  | *Bajra/Shishua** | Pearl millet | *Pennisetum typhoideum* Rich. | 348 | 11 | 61.78 | 5.43 | 11.49 | NA | NA | 0.25 | 0.2 | 6.42 | 2.76 | 27.35 | 36.11 | 289 |
|  | *Kodo/Mandua** | Finger millet | *Eleusine coracana* (L.) Gaertn. | 321 | 7.2 | 66.82 | 1.92 | 11.18 | 1.53 | NA | 0.37 | 0.17 | 4.62 | 2.53 | 364 | 34.66 | 210 |
|  | *Jowar** | Sorghum | *Sorghum vulgare* Pers. | 334 | 10 | 67.68 | 1.73 | 10.22 | 8.29 | NA | 0.35 | 0.14 | 3.95 | 1.96 | 27.6 | 39.42 | 274 |
|  | *Jatta Usro/Ghangra^#^* | Cowpea, brown | *Vigna catjang* (L.) Walp. | 365 | 24.2 | 61.9 | 2.3 | 28.6 | 10 | NA | 0.33 | 0.09 | 6.07 | 3.83 | 91 | 231 | NA |
|  | *Eso Usro** | Cowpea, white | *Dolichos catjang* Burm.f | 320 | 21.3 | 53.77 | 1.14 | 11.7 | 8.21 | NA | 0.33 | 0.09 | 5.04 | 3.57 | 84.1 | 249 | 378 |
|  | *Kakro/Suthro/*  *Suthri^$^* | Rice bean | *Phaseolus calcaratus* Roxb. | 332 | 21.5 | 60.9 | 0.3 | NA | NA | NA | NA | NA | NA | NA | 302 | NA | 297 |
|  | *Kurthi** | Horse Gram, whole | *Dolichos biflorus* L. | 321 | 22 | 57.2 | 0.5 | NA | 58.57 | NA | 0.32 | 0.24 | 8.76 | 2.71 | 269 | 163 | 298 |
|  | *Khesari dal^$^* | Khesari *Dal* | *Lathyrus sativus* L. | 345 | 28.2 | 56.6 | 0.6 | NA | NA | NA | NA | NA | 6.3 | NA | 90 | NA | 317 |
|  | *Makedi Ghasi ** | Colocasia leaves | *Colocasia antiquorum* Schott | 44 | 3.4 | 3.69 | 1.38 | 5.6 | 146 | 40.71 | 0.08 | 0.07 | 3.41 | 0.82 | 216 | 159 | 57.88 |
|  | *Daav Ghasi ^#^* | Kantha leaves | *Euphorbia granulate* Forssk. | 46 | 3.5 | 8 | NA | 7.1 | 11680 | 9 | 3.07 | NA | 81.09 | 1.01 | 425 | 7.2 | NA |
|  | *Lol Ghasi^$^* | Bottle gourd leaves | *Lagenaria vulgaris* Ser. | 39 | 2.3 | 6.1 | 0.7 | NA | NA | NA | NA | NA | NA | NA | 80 | NA | 59 |
|  | *Sanjhori Saag** | Drumstick leaves | *Moringa oleifera* Lam. | 67 | 6.4 | 5.62 | 1.64 | 8.21 | 17542 | 108 | 0.06 | 0.45 | 4.56 | 0.72 | 314 | 42.89 | 109 |
|  | *AdroGhasi/*  *Margi adro** | Amaranth leaves | *Amaranthus spinosus* L. | 24 | 1.6 | 2.01 | 0.45 | 2.21 | 1594 | 77.3 | 0.01 | 0.13 | 6.37 | 1.57 | 359 | 41.44 | 72.46 |
|  | *Gochi Ghasi** | Ponnaganni | *Alternanthera sessilis* (L.) R.Br. ex DC. | 51 | 5.3 | 5.17 | 0.71 | 6.74 | 5288 | 103 | 0.02 | 0.1 | 3.88 | 0.99 | 388 | 48.42 | 53.26 |
|  | *Kondi Ghasi ^#^* | Dhurup leaves | *Leucas lavandulifolia*  Sm. | 67 | 5.7 | 11.1 | NA | 6.7 | 18460 | 8 | NA | NA | 20.02 | 0.8 | 236 | 10.7 | NA |
|  | *Boot Ghasi ^$^* | Bengal gram leaves | *Cicer arietinum* L. | 97 | 7 | 14.1 | 1.4 | NA | NA | NA | NA | NA | 23.8 | NA | 340 | NA | 120 |
|  | *Pakedi Ghasi ^#^* | Banyan leaves | *Ficus benghalensis* L. | 121 | 2.9 | 27.3 | NA | 22.3 | 8200 | NA | NA | NA | 2.77 | 0.8 | 295 | 3.9 | NA |
|  | *Nasni Ghasi^* | Garlic leaves | *Allium sativum* L*.* | 34 | 3.1 | 5.4 | NA | 4.9 | 5100 | 6 | NA | NA | 5.95 | 0.21 | 221 | 2.9 | NA |
|  | *Chiniya saag^* | - | *Brassica campestris* L. | 31 | 1.5 | 6.3 | NA | 3.5 | 4290 | 11 | NA | 0.33 | 5.93 | NA | 274 | 0.96 | NA |
|  | *Bir Karela ** | Bitter gourd | *Momordica dioica* Roxb. | 19 | 1.3 | 2.53 | 0.24 | 3.49 | 126 | 50.87 | 0.06 | 0.04 | 1.08 | 0.36 | 16.27 | 51.45 | 40.21 |
|  | *Simbi^$^* | Field beans, tender | *Dolichos lablab* L. | 48 | 3.8 | 7.2 | 0.1 | NA | 187 | 9 | 0.1 | 0.06 | 0.83 | 0.4 | 210 | NA | NA |
|  | *Jhingli (Desi)** | Ridge gourd | *Luffa acutangula* (L.) Roxb. | 13 | 0.9 | 1.72 | 0.14 | 1.81 | 348 | 5.42 | 0.02 | 0.01 | 0.42 | 0.22 | 13.7 | 29.26 | 33.06 |
|  | *Maas ardo (baans)/Karu** | Bamboo, Tender | *Bambusa vulgaris* Schrad. | 16 | 1.3 | 1.67 | 0.35 | 1.55 | NA | 15.74 | 0.06 | 0.06 | 0.33 | 0.37 | 10 | 17.05 | 39.63 |
|  | *Zarkunda^$^* | Ash gourd | *Benincasa hispida* (Thunb.) Cogn. | 10 | 0.4 | 1.9 | 0.1 | NA | NA | 1 | 0.06 | 0.01 | 0.8 | NA | 30 | NA | 20 |
|  | *Kokri/*  *Kakori^$^* | Spine gourd | *Momordica dioica* Roxb. | 52 | 3.1 | 7.7 | 1 | 3 | NA | NA | NA | NA | 4.6 | NA | NA | NA | 42 |
|  | *Kundri** | Kovai | *Coccinia cordifolia* (L.) Cogn*.* | 19 | 1.2 | 2.41 | 0.24 | 3.25 | 147 | 21.08 | 0.04 | 0.02 | 0.29 | 0.13 | 37.12 | 50.13 | 26.29 |
|  | *Jatta Usro ^#^* | Barbatti vegetable | *Vigna catjang* (L.) Walp. | 49 | 3.7 | 8.5 | NA | 4.4 | 36 | 9 | NA | NA | 0.95 | 0.61 | 41 | 7 | NA |
|  | *Makedi (Alli)** | Colocasia | *Colocasia antiquorum* Schott | 90 | 3.3 | 17.85 | 0.17 | 3.22 | 6.5 | 1.83 | 0.06 | 0.03 | 0.66 | 0.41 | 30.18 | 19.91 | 81.16 |
|  | *Ambad Pupu^$^* | Ambada | *Spondias mangifera* Wild. | 48 | 0.7 | 4.5 | 3 | NA | NA | NA | NA | NA | 3.9 | NA | 36 | NA | 11 |
|  | *Madgi^$^* | Mahua, ripe | *Madhuca indica* J.F.Gmel. | 111 | 1.4 | 22.7 | 1.6 | NA | 307 | 40 | NA | NA | 0.23 | NA | 45 | NA | 22 |
|  | *Kero/Keero Toso^$^* | Marking nut (kernel) | *Semecarpus anacardium* L.f. | 587 | 26.4 | 28.4 | 36.4 | NA | NA | NA | NA | NA | 6.1 | NA | 295 | NA | NA |
|  | *Ber/Ilkarpu* | Zizyphus | *Zizyphus jujube* Mill. | 49 | 1.3 | 9.4 | 0.35 | 3.73 | 1.5 | 60.93 | 0.01 | 0.02 | 0.4 | 0.1 | 46.55 | 5.93 | 32.38 |
|  | *Telo/Kenda/ Kaanda^$^* | *Tumki* | *Diospyros melanoxylon* Roxb. | 112 | 0.8 | 26.8 | 0.2 | NA | 361 | 1 | 0.01 | 0.04 | 0.5 | NA | 60 | NA | NA |
|  | *Talmi Gheedi^$^* | Palmyra fruit, ripe (mesocarp) | *Borassus flabellifer* L. | 87 | 0.7 | 20.7 | 0.2 | NA | NA | NA | NA | NA | NA | NA | 9 | NA | NA |
|  | *Tamli** | Taal Phal | *Borassus flabellifer* L. | 101 | 0.5 | 4.92 | 0.12 | 2.4 | NA | 0.25 | 0.01 | NA | NA | 0.05 | NA | 24.4 | NA |
|  | *Pakkedi^$^* | Banyan fruit | *Ficus benghalensis* L. | 72 | 1.7 | 11.8 | 2 | NA | NA | NA | NA | NA | NA | NA | 364 | NA | NA |
|  | *Bel/Otte** | Wood apple | *Aegle marmelos* (L.) Correa | 136 | 2.6 | 28.21 | 0.57 | 6.31 | 2.5 | 7.5 | 0.03 | 0.04 | 0.23 | 0.14 | 47.95 | 55.22 | 37.29 |
|  | *Ghongri^$^* | Snail | *Pila globoasa* | 97 | 10.5 | 12.4 | 0.6 | NA | NA | NA | NA | NA | NA | NA | 870 | NA | 116 |
|  | *Potta Machhli^$^* | Puti | *Burbus* spp. | 106 | 18.1 | 3.1 | 2.4 | NA | NA | 15 | NA | NA | 1 | NA | 110 | NA | NA |
|  | *Singhi fish^$^* | Singhi | *Saccobranchus fossilis* | 124 | 22.8 | 6.9 | 0.6 | NA | NA | NA | NA | NA | 2.3 | NA | 670 | NA | NA |
|  | *Magur/Mangri^$^* | Walking catfish | *Clarias batrachus* | 86 | 15 | 4.2 | 1.0 | NA | NA | NA | NA | NA | 0.7 | NA | 210 | NA | 290 |
|  | *Boari^$^* | Wallago | *Wallago attu* | 116 | 15.4 | 7.6 | 2.7 | NA | NA | 8 | NA | NA | 1 | NA | 160 | NA | 490 |
|  | *Gacchi** | Freshwater Eel | *Anguilla Anguilla* | 108 | 20.4 | NA | 2.63 | NA | 866^a^ | NA | NA | 0.31 | 1.54 | 2.23 | 52.99 | 1294 | 361 |
|  | *Tengra/Tonger^$^* | Catfish | *Mystus vittatus* | 144 | 19.2 | 2.3 | 6.4 | NA | NA | 18 | NA | NA | 2 | NA | 270 | NA | 170 |
|  | *Silong** | Silhan | *Silonia silondia* | 156 | 22.7 | NA | 6.68 | NA | 3.07 ^a^ | NA | 0.02 | 0.07 | 0.56 | 0.58 | 28.82 | 2438 | 163 |
|  | *Baale^$^* | Bele fish | *Glossogoboius giuris* | 75 | 14.5 | 2.9 | 0.6 | NA | NA | 3 | NA | NA | 1 | NA | 370 | NA | 330 |
|  | *Maako/jhinuk^$^* | Mussel | *Margaritifera margaritifera* | 81 | 14.5 | 2.1 | 1.6 | NA | NA | NA | NA | NA | NA | NA | 592 | NA | NA |
|  | *Chetado ande^$^* | Eggs of red ants | *Aceophylla smaragdina* | 131 | 13.4 | 9.1 | 4.6 | NA | NA | NA | NA | NA | NA | NA | 104 | NA | 107 |
|  | *Moosa^$^* | Field Rat | *Rattus argentiventer* | 104 | 23.6 | NA | 1 | 0.1 | NA | NA | NA | NA | NA | NA | 30 | NA | NA |
|  | *Jangli Murgi^$^* | Fowl/Rooster | *Galloanserae* | 109 | 25.9 | NA | 0.6 | NA | NA | NA | NA | 0.14 | NA | NA | NA | 6.8 | NA |
|  | *Pervan** | Pigeon | *Columba livia domestica* | 126 | 17.9 | NA | 6.03 | NA | 15.25^a^ | NA | 0.18 | 0.39 | 3.81 | 2.48 | 18.11 | 8.41 | 255 |
|  | *Bater** | Quail | *Coturnix coturnix* | 138 | 20.9 | NA | 5.95 | NA | 12.84^a^ | NA | 0.05 | 0.24 | 1.90 | 1.13 | 20.60 | 9.33 | 300 |
|  | *Jangli suar/kissu** | Pig | *Sus scrofa* | 179 | 19.4 | NA | 11.3 | NA | 1.51^a^ | NA | 0.3 | 0.11 | 1 | 1.34 | 8.12 | 7.74 | 143 |

*Note*: Text in Italics represents Paharia

NA=Not available

^a^ Retinol expressed in µg/100gm for animal foods

*(Longvah et al. 2017)

^ (Ghosh-Jerath et al. 2015)

^#^ (Ghosh-Jerath, Singh, Magsumbol, Kamboj, et al. 2016)

^$^(Gopalan, Sastri, and Balasubramanian 1989)
